# Advanced slime mould algorithm incorporating differential evolution and Powell mechanism for engineering design

**DOI:** 10.1016/j.isci.2023.107736

**Published:** 2023-08-28

**Authors:** Xinru Li, Zihan Lin, Haoxuan Lv, Liang Yu, Ali Asghar Heidari, Yudong Zhang, Huiling Chen, Guoxi Liang

**Affiliations:** 1 Key Laboratory of Intelligent Informatics for Safety & Emergency of Zhejiang Province, Wenzhou University, Wenzhou 325035, China; 2 School of Surveying and Geospatial Engineering, College of Engineering, University of Tehran, Tehran, Iran; 3 School of Computing and Mathematical Sciences, University of Leicester, Leicester LE1 7RH, UK; 4 Department of Information Technology, Wenzhou Polytechnic, Wenzhou 325035, China

**Keywords:** Applied sciences, Engineering

## Abstract

The slime mould algorithm (SMA) is a population-based swarm intelligence optimization algorithm that simulates the oscillatory foraging behavior of slime moulds. To overcome its drawbacks of slow convergence speed and premature convergence, this paper proposes an improved algorithm named PSMADE, which integrates the differential evolution algorithm (DE) and the Powell mechanism. PSMADE utilizes crossover and mutation operations of DE to enhance individual diversity and improve global search capability. Additionally, it incorporates the Powell mechanism with a taboo table to strengthen local search and facilitate convergence toward better solutions. The performance of PSMADE is evaluated by comparing it with 14 metaheuristic algorithms (MA) and 15 improved MAs on the CEC 2014 benchmarks, as well as solving four constrained real-world engineering problems. Experimental results demonstrate that PSMADE effectively compensates for the limitations of SMA and exhibits outstanding performance in solving various complex problems, showing potential as an effective problem-solving tool.

## Highlights

• A new SMA-based method integrating DE and Powell mechanisms, named PSMADE, is proposed
• PSMADE effectively improves SMA performance on unimodal and multimodal functions
• PSMADE outperforms other high-performance optimizers on the CEC 2014 benchmark
• PSMADE successfully solves four real-world engineering problems

## Graphical abstract

**Figure:**
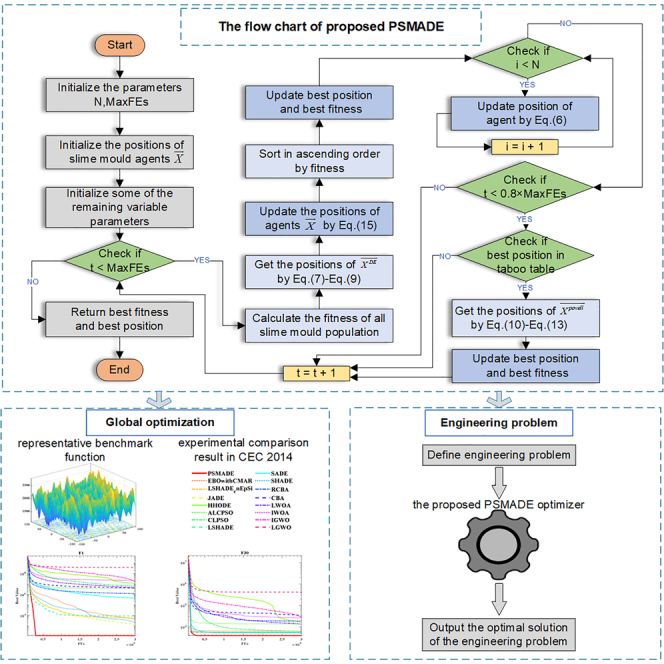

## Introduction

As technology advances rapidly, fundamental research in various fields of data-driven computational intelligence has deepened in recent years.^1^^,^^2^ The complexity of modern engineering operations can be a challenge for industry engineers looking to achieve high performance optimization algorithms, optimal results, or optimal scalability.^3^^,^^4^^,^^5^ While this task can be quite onerous, technological progressions in artificial intelligence offer invaluable resources in overcoming these obstacles.^4^^,^^6^ AI-based solutions can provide insight that allows engineers to make the most astute optimizations and maximize results.^7^^,^^8^ As AI continues to evolve, industry engineers will be able to build even more sophisticated strategies, helping them to optimize operations at a higher rate than ever before.^9^ Therefore, various complex, large-scale global optimization problems have emerged.^10^^,^^11^ When facing intricate optimization processes, deterministic techniques can occasionally prove to be insufficient, particularly in cases where the function is either non-differentiable or multimodal.^12^^,^^13^ Despite their value when looking for a rough or “satisfactory” solution, deterministic strategies will need to be used in combination with other approaches such as genetic algorithms, Monte Carlo simulations, or local search techniques to tackle more intricate challenges.^14^^,^^15^ Compared with mathematical methods and traditional optimization algorithms, nature-inspired metaheuristics algorithms (MAs) can somewhat leapfrog the vast complexity of mathematical reasoning, possible determinism, and other problems and are often leaders in treating complex issues in various fields.

Optimization problems come in all shapes and sizes, and they can be broken down into a number of different categories, such as robust optimization,^16^^,^^17^ evolutionary methods, and memetic methods,^18^ and in other way, multi- or many-objective or single objective problems, with the latter representing a specialized subset of previous ones. MAs are mainly divided into evolutionary algorithms and swarm intelligence optimization algorithms. Evolutionary algorithms are inspired by the laws of evolution in nature and achieve the overall progress of a population by simulating the competition and elimination behaviors among individuals of the population.^19^ Among them, the representative evolution algorithm includes the genetic algorithm (GA),^20^ differential evolution (DE),^21^ and evolution strategy (ES).^22^ Swarm intelligence algorithms simulate the social organization of a population of organisms and the cooperative behavior among individuals within the population to obtain a globally optimal solution. Among them, the typical swarm intelligence optimization algorithm includes ant colony optimization (ACO),^23^ particle swarm optimization (PSO),^24^ artificial bee colony (ABC),^25^ cuckoo search (CS),^26^ and firefly algorithm (FA).^27^ In addition, with the in-depth research of scholars on swarm intelligence optimization algorithms, new algorithms have been proposed continuously, including gray Wolf optimizer (GWO),^28^ sine cosine algorithm (SCA),^28^ whale optimization algorithm (WOA),^29^ salp swarm algorithm (SSA),^28^ harris hawks optimization (HHO) (https://aliasgharheidari.com/HHO.html),^30^ and slime mould algorithm (SMA) (https://aliasgharheidari.com/SMA.html),^31^^,^^32^ hunger games search (HGS) (https://aliasgharheidari.com/HGS.html),^33^ RUNge Kutta optimizer (RUN) (https://aliasgharheidari.com/RUN.html),^34^ colony predation algorithm (CPA),^35^ rime optimization algorithm (RIME) (https://aliasgharheidari.com/RIME.html)^36^ and weighted mean of vectors (INFO) (https://aliasgharheidari.com/INFO.html).^37^ These intelligent algorithms are simple in principle and easy to implement. They can effectively solve complex optimization problems, such as bankruptcy prediction,^38^ scheduling optimization,^39^ economic emission dispatch,^40^ numerical optimization,^41^^,^^42^^,^^43^ large-scale complex optimization,^44^ feedforward neural networks,^45^ feature selection,^46^^,^^47^^,^^48^^,^^49^^,^^50^^,^^51^ and multi-objective optimization.^52^

SMA is a metaheuristic algorithm based on the behavioral patterns of intelligent organisms in nature, which was proposed by Li et al.^53^ in 2020. SMA possesses various unique features and advantages, addressing complex problems through actively exploring the surrounding environment, exchanging information via chemical substances, and self-organizing approaches. The intelligence observed in nature inspires us to develop algorithms employing similar strategies to tackle problems that are complex and difficult to solve using traditional methods. Furthermore, SMA exhibits good adaptability and robustness. Organisms such as slime moulds can adjust their behavior and achieve optimal solutions based on environmental changes and goal variations. This makes SMA particularly suitable for problem spaces characterized by complexity and diversity. Additionally, SMA demonstrates high computational efficiency and parallelism. When searching for food and transmitting information, slime moulds can form efficient network structures through their branching structures and the diffusion of chemical substances. The efficient network structure of SMA facilitates rapid computation and effective parallelization, rendering it highly suitable for tackling complex problems on a large scale. These attributes make SMA a valuable tool across multiple domains. For instance, Khajavi et al.^54^ utilized a Random Forest model in combination with SMA (RF-SMA) to accurately predict carbon dioxide emissions resulting from road transport, demonstrating superior performance compared to the other seven groups of intelligent optimization algorithms evaluated in the enhanced random forest model. Eskandaripour et al.^55^ utilized the SMA to optimize low-impact development (LID), resulting in the creation of a novel stormwater management model (SWMM-SMA). This model effectively facilitates the design and control of optimal LID practices and improves runoff quality in urban areas. Chakraborty et al.^56^ effectively minimized operating costs in microgrids by utilizing the SMA algorithm, while Wu et al.^57^ achieved significant improvements in convergence speed and solution accuracy for optimizing truss structures through an improved version of the SMA algorithm compared to similar products.

However, according to the theory of “No Free Lunch” (NFL),^58^ no algorithm can achieve perfect optimization in any given domain. Therefore, although SMA is a competitive algorithm that performs well in certain situations, it also has limitations and shortcomings. Thus, it is imperative to integrate its principles and characteristics with corresponding measures to address these issues and fully exploit its capabilities. Achieving optimal performance of SMA poses a significant challenge in striking a balance between the exploration and exploitation stages.^59^ Overemphasis on exploration may lead to slower convergence, while overemphasis on exploitation may result in premature convergence toward suboptimal solutions. The equilibrium between these two phases largely determines an algorithm’s ability to explore novel solutions and optimize existing ones.^60^

To address this issue, extensive improvement studies have been conducted by relevant researchers, with a primary focus on two directions: enhancing the algorithmic mechanism and integrating it with other algorithms. The first is to improve the mechanism for SMA. For example, WQSMA proposed by Yu et al.^61^ combined a quantum rotation gate and water cycle operation mechanism. By introducing quantum dynamic selection, this algorithm can effectively explore feasible regions and mitigate the local optima issue using rotating gates and water cycle strategies, thereby maintaining a balance between exploration and exploitation tendencies. Hu et al.^62^ proposed a parameter improvement strategy, DFSMA, which integrates a dispersion foraging strategy and a novel dispersion degree into SMA. The former introduces stochasticity to the search process, facilitating the exploration of a broader solution space, while the latter enhances the distance utilization rate, effectively increasing the likelihood of discovering optimal solutions and accelerating algorithm convergence. Hu et al.^63^ proposed a hierarchical guided slime mould algorithm (HG-SMA) that employs distinct guidance strategies for different levels of individuals. The improvement involves dividing the population into elite and general groups, then applying the reciprocity and learning strategies to enhance their exploration and exploitation capabilities, respectively. Wu et al.^57^ proposed a Gaussian barebone mutation enhanced SMA, in which the incorporation of a Gaussian function not only accelerated the convergence speed but also expanded the search space. Additionally, the introduction of the DE update strategy enhances global search performance to some extent.

The second is to integrate with other algorithms. For example, Ewees et al.^59^ have combined SMA with gradient-based optimizer (GBO) and introduced SMA as the local search strategy of GBO to enhance the algorithm’s exploration ability in the search space, leveraging both approaches to their fullest potential. Abdel-Basset et al.^64^ proposed an enhanced algorithm (HSMA_WOA) that integrates the WOA and SMA. This integration effectively balances exploration and exploitation by leveraging WOA’s ability to identify potentially feasible regions and SMA’s capacity for local development. Deng et al.^65^ proposed an enhanced SMA (MSMA) that integrates the mutation strategy of DE, dynamic random search technology, and adaptive mutation. The former effectively balances exploration and exploitation, while the latter maintain population diversity to some extent and encourages the method to escape from local optima by adapting its mutation rate according to the current state of convergence.

Despite previous efforts to improve the balance between exploration and exploitation in SMA, many of these strategies may still exhibit bias toward one another, resulting in an unstable equilibrium that leads to prolonged search times or poor convergence. To enhance the adequate balance between exploration and exploitation of SMA, we have addressed the issues of low convergence accuracy, slow convergence speed, and susceptibility to local optima by integrating the DE mechanism with Powell’s mechanism,^66^ resulting in our proposed PSMADE algorithm. First, we have incorporated the DE mechanism into SMA, leveraging the best positions found by each population member to exchange information and effectively utilizing the knowledge of exceptional individuals within the population to expedite convergence toward a globally optimal solution. Additionally, the differential mutation operation in the DE mechanism enhances population diversity, enabling the algorithm to escape local optima and discover superior solutions. Meanwhile, we have incorporated the Powell mechanism into the PSMADE algorithm to enhance its search capability through local exploration and iterative refinement, enabling it to approximate the desired solution more accurately.

Compared to traditional and other improved algorithms, PSMADE exhibits significant advantages across multiple aspects. First, the proposed algorithm significantly improves both convergence accuracy and speed. A DE mechanism allows for quicker identifying regions near-optimal solutions, while the Powell mechanism refines solution accuracy through local search. Second, the differential mutation operation and population information exchange effectively mitigate the issue of local optima in PSMADE, while maintaining population diversity during the search process to increase the likelihood of discovering superior solutions.

In this study, we conducted comparative experiments on 30 benchmark test problems from CEC 2014 benchmark and utilized the Wilcoxon signed-rank test^67^ for comprehensive analysis to demonstrate that PSMADE exhibits superior convergence speed, accuracy, and capability in solving complex optimization problems. Moreover, to further evaluate the feasibility of PSMADE, we have applied it to solve four real-world engineering problems with constraints. This demonstrates that PSMADE performs exceptionally well on benchmark problems and showcases its strong adaptability and practical value in addressing real-world issues. The contributions and highlights of this paper are as follows.

• The DE mechanism is integrated to update the particle positions by utilizing information from superior individuals, enhancing the algorithm’s global exploration capability and mitigating premature convergence issues.
• The Powell mechanism and taboo table have been incorporated to enhance the algorithm’s capacity for exploiting local regions and achieving superior convergence toward the target solution.
• The optimization performance of PSMADE has been validated through comparison with 14 well-known optimizers and 15 advanced improved algorithms on CEC 2014 benchmark.
• The PSMADE algorithm has demonstrated its effectiveness and feasibility in practical applications by successfully solving four real-world engineering problems.

## Results and discussion

### Qualitative analysis of PSMADE

**Figure 1:**
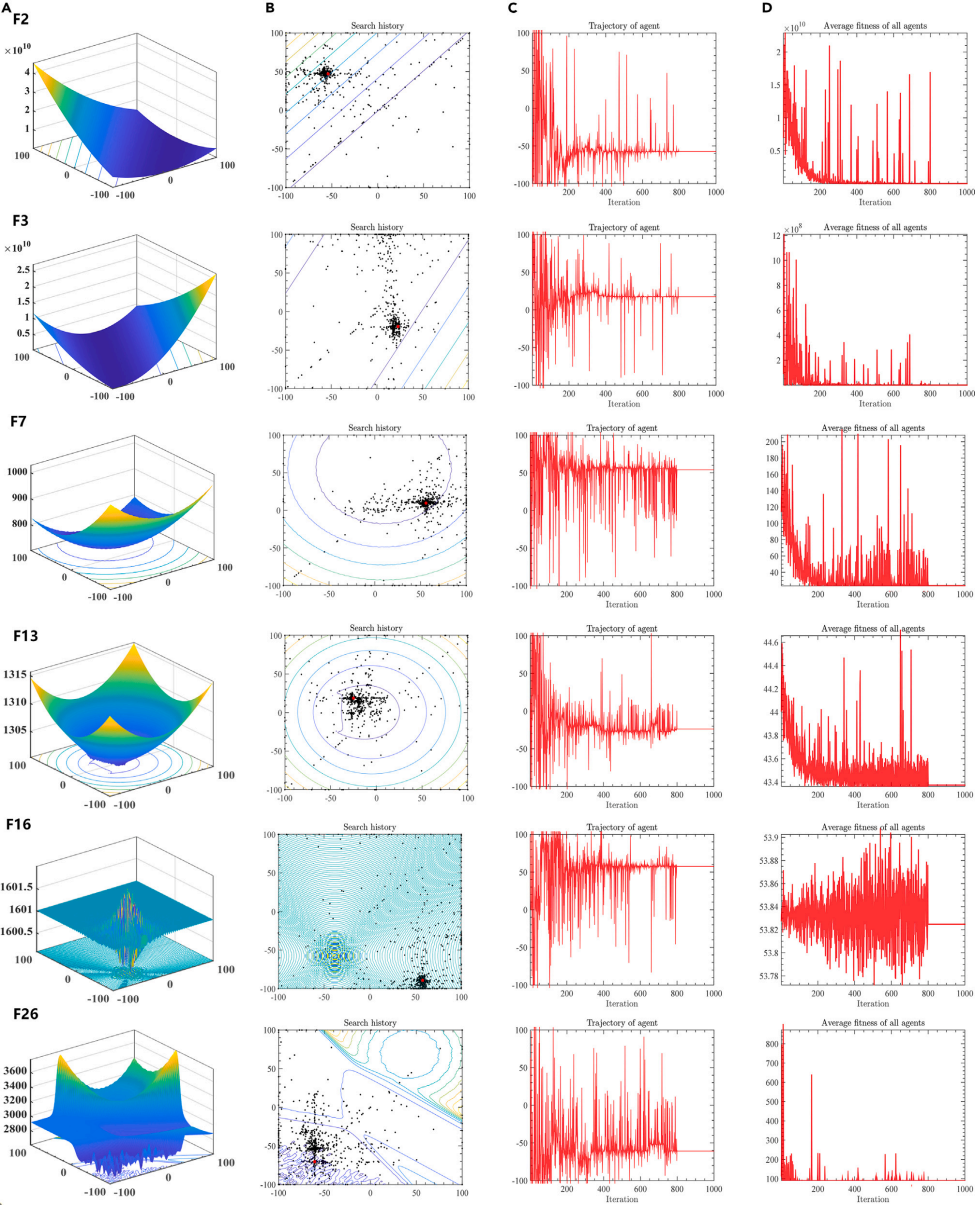
The history trajectory analysis for PSMADE (A–D) (A) Graphical plots of functions; (B) Search history of PSMADE; (C) Trajectory of PSMADE in the first dimension; (D) The average fitness of PSMADE.

This subsection presents a qualitative analysis of the proposed PSMADE, demonstrating its performance on various types of functions through graphical representations to provide insights into the exploration and exploitation phases. Figure 1 showcases the qualitative results of PSMADE in unimodal, multimodal, and hybrid functions by analyzing representative evaluation functions from test experiments. Among them, Figure 1A graph displays the 3D positional distribution of the function, while Figure 1B graph illustrates the 2D position and distribution of search history for slime mould population in PSMADE, reflecting their evolutionary process and distribution. The Figure 1C chart depicts the trajectory of PSMADE, where individual slime moulds’ movement trajectories reveal changing patterns in one-dimensional space. Figure 1D displays the average fitness value of the slime mould population throughout the PSMADE search process, illustrating its changing trend with each iteration.

The optimal global solution of the optimization problem is indicated by the red dots in Figure 1B, while the distribution of black dots clearly depicts the search trajectories of slime mould members. The distribution of black points and the position of red points in Figure 1B demonstrate that individuals in the PSMADE algorithm gradually converge toward the optimal solution, resulting in fewer iterations to find the global optimum for unimodal, multimodal, or hybrid functions. Moreover, figures F3, F7, F13, and F26 demonstrate the PSMADE algorithm’s ability to evade local optima despite having multiple optimal values. Additionally, upon examining the trajectory of the PSMADE algorithm depicted in Figure 1C, it is apparent that the slime mould individual exhibits a high vibration amplitude during the initial search phase, which gradually decreases with an increase in iterations until it stabilizes. This phenomenon suggests that slime mould individuals continuously explore the global optimal solution in the early stages. In contrast, in later stages, they primarily engage in local exploration around the optimal solution to avoid getting trapped in a local optimum situation. This further demonstrates the high adaptability and robustness of the PSMADE algorithm to various types of problems. The average fitness value of the slime mould population during the search process, as shown in Figure 1D, provides essential information, and its changing trend with iteration reflects the performance and effectiveness of this algorithm in solving optimization problems. The population’s mean fitness value gradually declines with each iteration, indicating significant progress in the algorithm’s pursuit of an optimal solution.

**Figure 2:**
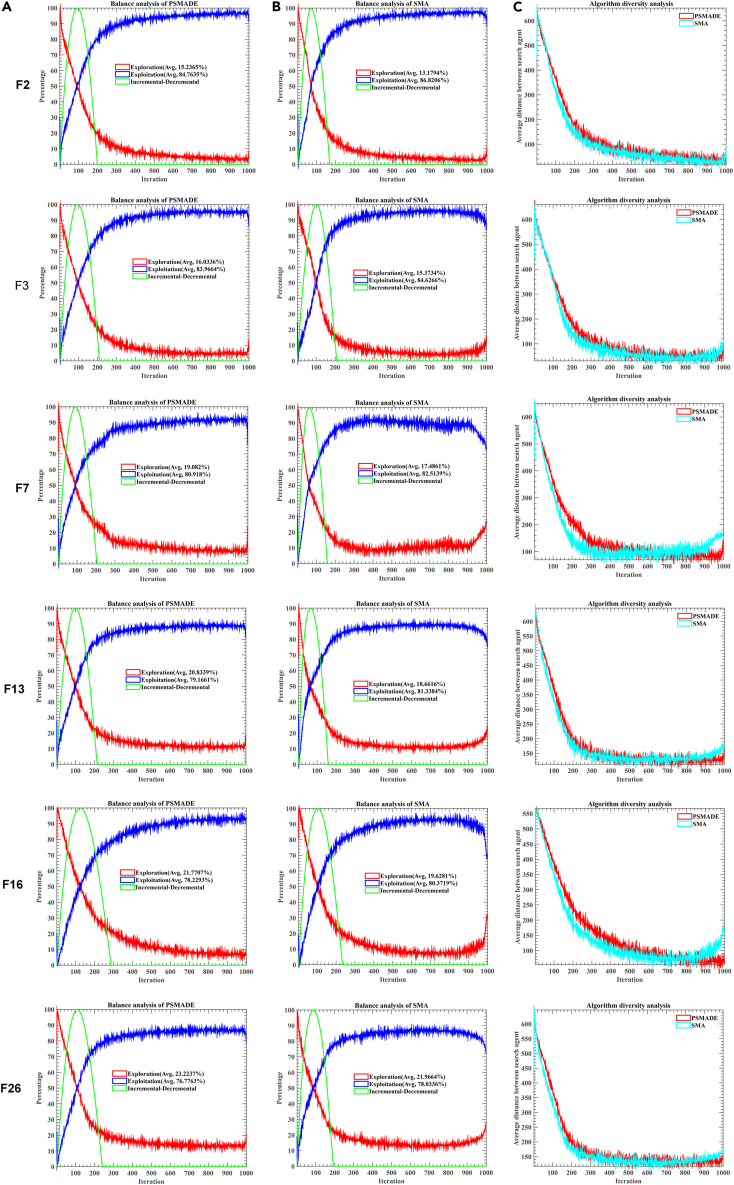
The balance and diversity analysis for PSMADE and SMA (A–C) (A) Balance analysis of PSMADE; (B) Balance analysis of SMA; (C) Diversity analysis of PSMADE and SMA.

To enhance the performance of PSMADE in global optimization problems, we conducted an empirical analysis to evaluate its exploration and exploitation capabilities compared to the original SMA algorithm. Figure 2A presents the balance analysis results of the PSMADE algorithm, while Figure 2B displays those of the SMA algorithm. However, Figure 2C illustrates the diversity analysis results of PSMADE and SMA algorithms. To visualize these results, an increase-decrease curve represents algorithmic balance and diversity. In this curve, when the global search result equals or exceeds the local search result, an upward trend is observed; conversely, a downward trend is displayed. When the curve value is negative, it is truncated to 0. High values in the curve indicate extensive exploration activities, while low values suggest more substantial exploitation effects. The duration of the curve reflects the continuous results of global or local search in the algorithm’s strategy. In Figure 2C, diversity analysis plots the number of iterations on the X axis and diversity measure on the Y axis, providing an intuitive representation of algorithmic performance.

The original SMA employs the weight attribute to adopt positive and negative feedback in response to environmental changes, resulting in superior performance in balancing global exploration and local exploitation. By examining Figures 2A and 2B, it is evident that the enhanced PSMADE algorithm not only inherits the exceptional performance of SMA but also further enhances the global promotion balance, thereby effectively improving SMA’s exploration ratio. The increased exploration ratio enables PSMADE to prioritize the development of novel solutions in the early stages and avoid being trapped in local optima through exploratory operations, thereby enhancing the likelihood of discovering global optimal solutions.

Moreover, as depicted in Figure 2C, it is evident that although the convergence rate of PSMADE’s diversity curve is slower than that of the original SMA, its ultimate convergence level surpasses that of the original SMA. The superiority of PSMADE over SMA lies primarily in its broader search space and higher-quality solutions. Firstly, in the initial exploration stage, PSMADE exhibits greater diversity than the original SMA, enabling it to explore a more comprehensive solution space and thus increasing the likelihood of discovering more potential optimal solutions. Secondly, through its comprehensive search of the solution space, PSMADE can converge toward superior solutions. Although PSMADE may require more iterations than the original algorithm, it is a worthwhile investment because it can increase the exploration ratio and expand search solution space, leading to the earlier discovery of superior solutions.

### Verification of the mechanisms

We have implemented a series of measures to ensure the objectivity and impartiality of experimental results. First, all experiments were conducted under standardized hardware conditions, utilizing Windows 10 Version 21H1 as the operating system, 64 GB RAM, and AMD Ryzen 7 5800H with Radeon Graphics (3.20 GHz) for programming purposes. Standardizing these hardware configurations ensures that all experiments are executed with equivalent computational resources, thereby avoiding results discrepancies due to software or hardware environment variations. Second, we have standardized the parameter settings of the metaheuristic algorithm by setting the population size to 30 and unifying the maximum number of evaluations to 300,000. This ensures all algorithms operate under identical search parameters, avoiding potential results bias due to different parameter settings. Additionally, to mitigate the impact of randomness on experimental outcomes, each algorithm was tested 30 times for every test function. Repeated trials smooth out random fluctuations within a single experiment and yield more stable performance evaluation results.

On the other hand, we employ the CEC 2014 benchmark as our testing dataset to assess the performance of PSMADE. This benchmark comprises 30 functions categorized into four types: unimodal (F1-F3), multimodal (F4-F16), hybrid (F17-F22), and composition (F23-F30) functions. A fair comparison is a required concern to be taken into consideration in computational science because if the comparison of several methods is on the wrong ground (not the same conditions), the evaluation will be invalid.^68^^,^^69^^,^^70^ By adopting the CEC 2014 benchmark, we can conduct a fair comparison and performance testing of different algorithms, providing a valid, reliable, and comprehensive evaluation of PSMADE’s performance.

To determine their performance, we comprehensively evaluate the experimental results obtained from the selected algorithms based on their average (Avg) and standard deviation (Stdv) of optimal function values. The best result for each problem is highlighted in bold font. The average value is a key index for measuring solution quality, with lower values indicating better global optimization search ability and solution quality. The standard deviation measures the degree of dispersion in the solution, and a lower standard deviation indicates greater algorithmic stability. During the evaluation, we utilized the non-parametric statistical test Wilcoxon signed-rank test to determine whether the improved method exhibited statistically significant differences with a significance level of 0.05. “+” indicates superiority over other methods, “ = ” denotes equivalence to other methods, and “-” signifies inferiority to other methods.

The proposed PSMADE integrates the DE mechanism and Powell mechanism into the original SMA to enhance its performance. To evaluate the effectiveness of these two strategies and their combination, we introduce three variants of SMA, as shown in Table 1. The naming convention for these variants includes “DE” to indicate integration with the DE mechanism and “P” to indicate integration with the Powell mechanism. Additionally, the data presented in Table 1 utilizes “T” and “F” to indicate whether or not the SMA has implemented the specified strategy.

**Table 1:** Various SMA variants with two strategies

mechanism	SMA	PSMA	SMADE	PSMADE
P	F	T	F	T
DE	F	F	T	T

**Figure 3:**
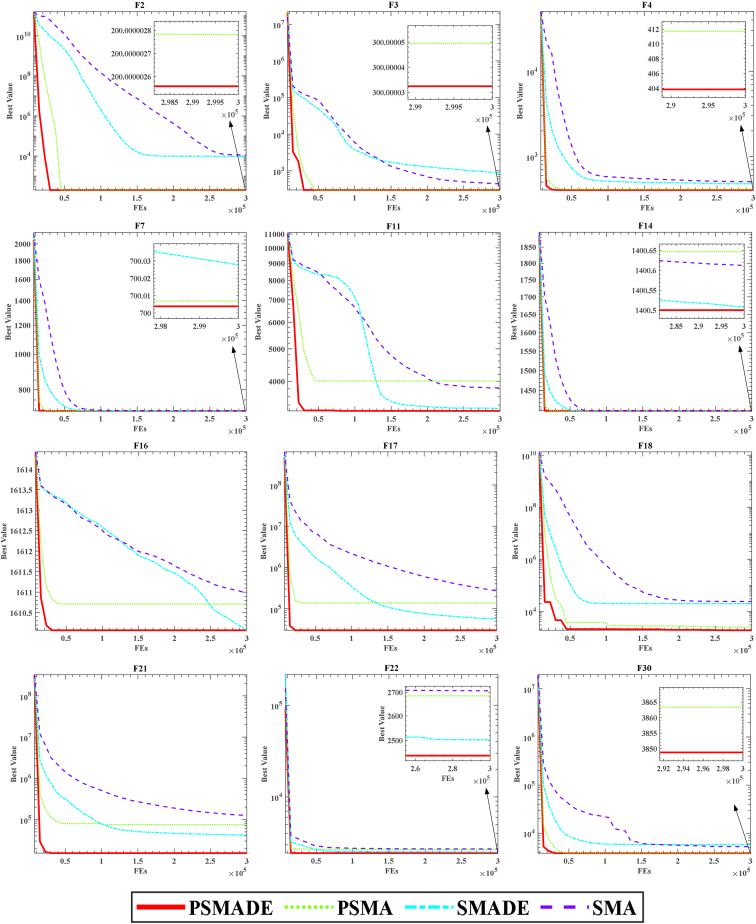
Fitness Convergence Comparison of PSMADE, PSMA, SMADE, and SMA on CEC 2014 Benchmark

The specific results of the experiments comparing the above three variants are shown in Table 2. The data can easily see in Table 2 that PSMADE excels on 16 of the 30 benchmark test functions, namely F1, F2, F4, F5, F6, F9, F15, F19, F23, F24, F25, F26, F27, F28, F29, and F30. Table 3 presents the results obtained from the Wilcoxon signed-rank test comparing the above algorithms with individual algorithm’s average ranking (ARV). Among them, PSMADE achieved the lowest ARV value of 1.366667 and ranked first. PSMA and SMADE obtained ARV values of 2.6 and 2.03333, respectively, which are higher than the ARV of the original SMA, indicating the effectiveness of the Powell and DE mechanisms. Additionally, we present the convergence curves of the aforementioned four algorithms, which provide a more intuitive demonstration of PSMADE’s performance on the CEC 2014 benchmark, as depicted in Figure 3. Based on the observations in Figure 3, incorporating the Powell mechanism has significantly improved convergence speed and accuracy for the algorithm. Additionally, introducing the DE mechanism has enhanced diversity, enabling it to overcome local optima more effectively than SMA alone. As a result, PSMADE exhibits superior convergence accuracy compared to PSMA and a faster convergence rate than SMADE.

**Table 2:** Results of various SMA on CEC 2014 benchmark

Function	Metric	PSMADE	PSMA	SMADE	SMA
F1	Avg	3.67796E+02	**1.03750E+02**	8.92800E+05	1.93634E+06
	Std	9.54812E+02	**1.41703E+01**	4.14692E+05	6.44363E+05
F2	Avg	2.00000E+02	**2.00000E+02**	9.65567E+03	1.13800E+04
	Std	5.94906E-07	**8.14380E-07**	1.20277E+04	1.26061E+04
F3	Avg	**3.00000E+02**	3.00000E+02	8.82928E+02	4.40950E+02
	Std	**1.77581E-04**	2.70937E-04	1.03520E+03	1.76359E+02
F4	Avg	**4.03845E+02**	4.11714E+02	4.78122E+02	5.03770E+02
	Std	**1.27344E+01**	2.25844E+01	3.27474E+01	3.70474E+01
F5	Avg	5.20000E+02	**5.20000E+02**	5.20893E+02	5.20868E+02
	Std	1.33826E-04	**4.12391E-04**	1.32491E-01	1.37312E-01
F6	Avg	6.09424E+02	6.16047E+02	**6.08342E+02**	6.14477E+02
	Std	3.31931E+00	3.20772E+00	**2.93118E+00**	3.65335E+00
F7	Avg	**7.00004E+02**	7.00007E+02	7.00028E+02	7.00272E+02
	Std	**6.15664E-03**	9.63443E-03	1.28211E-02	1.07249E-01
F8	Avg	8.04643E+02	8.13034E+02	**8.03422E+02**	8.14229E+02
	Std	2.01266E+00	3.68917E+00	**1.53831E+00**	3.96385E+00
F9	Avg	9.55320E+02	9.95184E+02	**9.51520E+02**	9.93316E+02
	Std	1.24588E+01	1.95797E+01	**1.75943E+01**	2.61887E+01
F10	Avg	1.18365E+03	1.60761E+03	**1.16167E+03**	1.56046E+03
	Std	1.11033E+02	2.42045E+02	**1.15229E+02**	2.52773E+02
F11	Avg	**3.26507E+03**	4.00821E+03	3.32122E+03	3.82343E+03
	Std	**6.31422E+02**	6.05832E+02	5.07135E+02	6.28467E+02
F12	Avg	1.20030E+03	1.20043E+03	**1.20013E+03**	1.20026E+03
	Std	1.01512E-01	1.76050E-01	**4.70379E-02**	8.78330E-02
F13	Avg	1.30032E+03	1.30047E+03	**1.30031E+03**	1.30046E+03
	Std	7.40669E-02	1.11146E-01	**7.37707E-02**	9.53696E-02
F14	Avg	**1.40050E+03**	1.40065E+03	1.40051E+03	1.40061E+03
	Std	**2.18138E-01**	3.31585E-01	2.19048E-01	3.56871E-01
F15	Avg	**1.50320E+03**	1.50584E+03	1.50373E+03	1.50631E+03
	Std	**8.89625E-01**	1.61613E+00	8.41873E-01	1.74437E+00
F16	Avg	**1.61007E+03**	1.61071E+03	1.61007E+03	1.61097E+03
	Std	**7.64724E-01**	5.89353E-01	6.51065E-01	6.07107E-01
F17	Avg	**2.99911E+04**	1.38392E+05	5.68498E+04	2.71490E+05
	Std	**2.38940E+04**	7.36637E+04	2.61671E+04	1.40555E+05
F18	Avg	**1.88090E+03**	2.47457E+03	1.99334E+04	2.39494E+04
	Std	**1.02595E+02**	8.31712E+02	8.66481E+03	6.03455E+03
F19	Avg	**1.90560E+03**	1.91530E+03	1.90566E+03	1.91520E+03
	Std	**1.50750E+00**	1.82890E+01	1.21456E+00	2.00705E+01
F20	Avg	2.17031E+03	2.19874E+03	**2.15987E+03**	2.18769E+03
	Std	1.37458E+02	1.41094E+02	**9.87160E+01**	6.49821E+01
F21	Avg	**1.52560E+04**	7.49360E+04	4.18411E+04	1.24484E+05
	Std	**1.46049E+04**	5.73891E+04	2.90459E+04	5.96762E+04
F22	Avg	**2.43976E+03**	2.68506E+03	2.50255E+03	2.70691E+03
	Std	**1.63430E+02**	1.71540E+02	1.61430E+02	2.21725E+02
F23	Avg	2.50000E+03	**2.50000E+03**	**2.50000E+03**	**2.50000E+03**
	Std	0.00000E+00	**0.00000E+00**	**0.00000E+00**	**0.00000E+00**
F24	Avg	2.60000E+03	**2.60000E+03**	**2.60000E+03**	**2.60000E+03**
	Std	0.00000E+00	**0.00000E+00**	**0.00000E+00**	**0.00000E+00**
F25	Avg	**2.70000E+03**	**2.70000E+03**	**2.70000E+03**	**2.70000E+03**
	Std	**0.00000E+00**	**0.00000E+00**	**0.00000E+00**	**0.00000E+00**
F26	Avg	**2.70026E+03**	2.70053E+03	2.70033E+03	2.70053E+03
	Std	**6.22640E-02**	1.18400E-01	6.20651E-02	1.21083E-01
F27	Avg	**2.90000E+03**	**2.90000E+03**	**2.90000E+03**	**2.90000E+03**
	Std	**0.00000E+00**	**0.00000E+00**	**0.00000E+00**	**0.00000E+00**
F28	Avg	**3.00000E+03**	**3.00000E+03**	**3.00000E+03**	**3.00000E+03**
	Std	**0.00000E+00**	**0.00000E+00**	**0.00000E+00**	**0.00000E+00**
F29	Avg	3.40563E+03	**3.12307E+03**	4.40588E+03	4.10350E+03
	Std	4.93911E+02	**3.38698E+01**	9.10080E+02	1.11863E+03
F30	Avg	**3.84879E+03**	3.86336E+03	5.80330E+03	5.25898E+03
	Std	**2.85795E+02**	4.23969E+02	1.04611E+03	1.62124E+03

**Table 3:** Average ranking values of involved algorithms by Wilcoxon signed-rank test

Algorithm	Rank	+/ = /-	ARV
PSMADE	1	∼	1.366667
PSMA	3	17/12/1	2.6
SMADE	2	13/15/2	2.033333
SMA	4	22/8/0	3

In summary, the DE and Powell mechanisms have proven to be effective in improving SMA. However, PSMADE, which combines the Powell and DE mechanisms, outperforms the other three mechanisms in terms of comprehensive performance.

### Comparison with conventional metaheuristic algorithms

In this section, we compare the proposed PSMADE with 12 well-known MAs on the CEC 2014 benchmark, including PSO,^71^ WOA,^72^ ABC,^25^ GWO,^73^ SSA,^74^ CS,^26^ MFO,^75^ HHO,^30^ BA,^76^ SCA,^77^ JAYA,^78^ TLBO,^79^ DE,^21^ and SMA.^53^ The parameter settings for all the conventional MAs involved in this experiment are listed in Table 4.

**Table 4:** Parameter setting of the comparison conventional algorithms with PSMADE

Algorithm	Other parameters
PSMADE	$z=0.03;F=\left[0.4,1\right];P_{CR}=\left[0.5,1\right]$
PSO	$c_{1}=2;c_{2}=\left[2,0\right];w=1;V_{\max}=6$
WOA	$b=1;a_{1}=\left[2,0\right];a_{2}=\left[-2,-1\right]$
ABC	$Number=\frac{N}{2};Limit=300$
GWO	a∈[0,2]
SSA	$c_{1}=\left[0,1\right];c_{2}=\left[0,1\right]$
CS	Pa=0.25;β=1.5
MFO	a∈[−1,−2];b=1;t=[−1,1]
HHO	$E_{0}=\left[-1,1\right];E_{1}=\left[2,0\right]$
BA	r=0.5;A=0.5
SCA	A=2
JAYA	∼
TLBO	TF={1,2}
DE	$F=\left[0.2,0.8\right],P_{CR}=0.2$
SMA	z=0.03

Based on the parameter settings mentioned above, Table 5 presents the specific results of a comparison experiment between PSMADE, an improved algorithm, and 14 conventional MAs on the CEC 2014 benchmark. The experimental data in Table 5 demonstrate that PSMADE performs well on 16 benchmark functions: F1, F2, F4, F5, F6, F9, F15, F19, F23-F30. The computed values of Avg and Stdv obtained from the experiments outperform those of the comparison algorithms in all experiments. Moreover, the experimental results demonstrate that PSMADE exhibits remarkable robustness in tackling diverse, complex problems, including unimodal functions F1 and F2, multimodal functions F4, F5, F6, F9, F15, and hybrid functions F19. Additionally, it outperforms other algorithms that compare composition functions, such as F23 to F30. In Table 6, we utilize the symbol “+/ = /-” to denote PSMADE’s overall performance on the CEC 2014 benchmark as superior, equivalent, or inferior to other algorithms and compute each algorithm’s average ranking. From the results, the ARV value of PSMADE algorithm is 2.0, ranking first. The ARV value of SMA algorithm is 4.966667, ranking fourth.

**Table 5:** Results of PSMADE and conventional algorithms on CEC 2014 benchmark

Function	Metric	PSMADE	PSO	WOA	ABC	GWO	SSA	CS	MFO
F1	Avg	**1.09857E+02**	8.44308E+06	2.82857E+07	4.49910E+06	5.37577E+07	1.75614E+06	1.34063E+06	1.19544E+08
	Std	**3.47205E+01**	2.43315E+06	1.10056E+07	1.76951E+06	3.51812E+07	7.10424E+05	6.69876E+05	1.48449E+08
F2	Avg	**2.00000E+02**	1.45557E+08	3.46213E+06	4.06387E+02	1.96064E+09	1.17511E+04	1.00000E+10	1.29836E+10
	Std	**6.71055E-07**	2.04638E+07	2.58424E+06	2.08751E+02	2.09879E+09	9.47260E+03	0.00000E+00	9.19373E+09
F3	Avg	3.00000E+02	9.66354E+02	3.46232E+04	1.32118E+03	3.01910E+04	1.51469E+03	3.00000E+02	9.82396E+04
	Std	9.24956E-09	1.23571E+02	2.04008E+04	9.28340E+02	7.84123E+03	7.59189E+02	3.94370E-09	5.50426E+04
F4	Avg	**4.06377E+02**	4.69638E+02	5.89057E+02	4.29530E+02	6.41295E+02	4.73768E+02	4.17366E+02	1.47919E+03
	Std	**1.70664E+01**	3.23847E+01	5.53157E+01	2.95211E+01	6.28318E+01	4.11092E+01	2.91772E+01	1.17309E+03
F5	Avg	**5.20000E+02**	5.20934E+02	5.20263E+02	5.20198E+02	5.20939E+02	5.20041E+02	5.20829E+02	5.20318E+02
	Std	**1.04272E-04**	4.69473E-02	1.62726E-01	3.18884E-02	4.61491E-02	7.81237E-02	6.96997E-02	1.75812E-01
F6	Avg	**6.09132E+02**	6.23097E+02	6.35474E+02	6.14367E+02	6.13366E+02	6.18366E+02	6.24669E+02	6.24275E+02
	Std	**3.46776E+00**	3.84859E+00	3.01073E+00	1.29815E+00	2.52994E+00	4.18471E+00	1.13242E+00	3.45118E+00
F7	Avg	7.00004E+02	7.02245E+02	7.01013E+02	7.00000E+02	7.16400E+02	7.00013E+02	7.00000E+02	8.07355E+02
	Std	6.86921E-03	1.57800E-01	6.59499E-02	3.13368E-04	1.53507E+01	1.31735E-02	3.60296E-05	5.70506E+01
F8	Avg	8.04079E+02	9.81140E+02	9.89006E+02	**8.00000E+02**	8.77513E+02	8.99871E+02	8.29752E+02	9.51357E+02
	Std	1.28935E+00	2.01216E+01	4.79795E+01	**7.89906E-14**	1.76294E+01	3.74027E+01	6.18174E+00	3.95590E+01
F9	Avg	**9.52998E+02**	1.10743E+03	1.13953E+03	9.84401E+02	9.95466E+02	1.02091E+03	1.03875E+03	1.12303E+03
	Std	**1.02268E+01**	2.82598E+01	6.22604E+01	1.35584E+01	1.70199E+01	3.15839E+01	2.75233E+01	5.99591E+01
F10	Avg	1.21711E+03	5.15048E+03	5.06382E+03	**1.00036E+03**	3.30125E+03	4.37991E+03	2.17670E+03	4.71821E+03
	Std	1.25352E+02	6.30131E+02	6.74491E+02	**3.41066E-01**	4.74567E+02	6.41350E+02	2.12701E+02	8.32531E+02
F11	Avg	3.29751E+03	5.89169E+03	6.09907E+03	**3.14870E+03**	3.77898E+03	4.99703E+03	4.57641E+03	5.23141E+03
	Std	5.33234E+02	5.08321E+02	8.13424E+02	**2.16964E+02**	6.10151E+02	6.46073E+02	2.13404E+02	8.24031E+02
F12	Avg	1.20032E+03	1.20246E+03	1.20181E+03	**1.20024E+03**	1.20171E+03	1.20044E+03	1.20075E+03	1.20048E+03
	Std	1.38735E-01	2.92502E-01	4.87165E-01	**3.93449E-02**	1.16069E+00	2.18483E-01	1.10671E-01	2.64946E-01
F13	Avg	1.30028E+03	1.30035E+03	1.30052E+03	**1.30023E+03**	1.30040E+03	1.30050E+03	1.30034E+03	1.30204E+03
	Std	5.92166E-02	8.07511E-02	9.53930E-02	**3.48316E-02**	1.00215E-01	1.38241E-01	5.25480E-02	1.19740E+00
F14	Avg	1.40044E+03	1.40029E+03	1.40026E+03	**1.40019E+03**	1.40449E+03	1.40033E+03	1.40024E+03	1.43555E+03
	Std	2.22315E-01	1.06140E-01	4.95625E-02	**1.75092E-02**	6.65951E+00	1.96195E-01	4.08086E-02	2.26021E+01
F15	Avg	**1.50339E+03**	1.51673E+03	1.58246E+03	1.50763E+03	1.58207E+03	1.50796E+03	1.51100E+03	1.97857E+05
	Std	**7.83495E-01**	1.32265E+00	3.37166E+01	8.27346E-01	2.39195E+02	2.98814E+00	1.58616E+00	4.08076E+05
F16	Avg	1.61008E+03	1.61210E+03	1.61259E+03	**1.60981E+03**	1.61085E+03	1.61155E+03	1.61233E+03	1.61273E+03
	Std	9.34915E-01	4.36227E-01	5.06985E-01	**4.17932E-01**	6.64469E-01	5.93497E-01	2.84228E-01	4.06264E-01
F17	Avg	2.16208E+04	2.94362E+05	3.38542E+06	2.26666E+06	1.44328E+06	1.36419E+05	**3.87004E+03**	3.62723E+06
	Std	1.97515E+04	1.43343E+05	2.40148E+06	8.33199E+05	2.35936E+06	1.05552E+05	**3.10196E+02**	5.88362E+06
F18	Avg	1.87316E+03	2.16264E+06	7.89879E+03	2.48588E+03	5.27485E+06	6.77711E+03	**1.87130E+03**	2.64746E+07
	Std	1.59078E+02	7.28018E+05	1.34875E+04	4.02626E+02	1.45461E+07	5.98363E+03	**1.88573E+01**	9.97564E+07
F19	Avg	**1.90615E+03**	1.91747E+03	1.94240E+03	1.90710E+03	1.94457E+03	1.91396E+03	1.90821E+03	1.98008E+03
	Std	**1.20175E+00**	2.80257E+00	3.90750E+01	7.98721E-01	2.66201E+01	2.17681E+00	8.75813E-01	8.12316E+01
F20	Avg	2.14343E+03	2.30736E+03	2.91137E+04	8.96765E+03	1.85622E+04	2.35219E+03	**2.05944E+03**	6.56586E+04
	Std	1.39649E+02	6.23234E+01	1.93601E+04	2.82234E+03	1.19928E+04	7.73988E+01	**1.72969E+01**	4.41141E+04
F21	Avg	1.93793E+04	1.26178E+05	1.54397E+06	2.12349E+05	1.16711E+06	6.14462E+04	**3.12552E+03**	9.23990E+05
	Std	2.36982E+04	7.64836E+04	1.97010E+06	1.02565E+05	2.21866E+06	4.40992E+04	**2.15830E+02**	2.36268E+06
F22	Avg	2.47684E+03	2.88678E+03	3.01178E+03	2.46069E+03	2.53766E+03	2.59391E+03	2.45174E+03	2.97672E+03
	Std	1.57688E+02	2.33664E+02	2.39437E+02	8.45668E+01	1.37195E+02	1.45922E+02	9.73843E+01	3.44090E+02
F23	Avg	2.50000E+03	2.61599E+03	2.63588E+03	2.61539E+03	2.63188E+03	2.61531E+03	2.61524E+03	2.66442E+03
	Std	0.00000E+00	4.74172E-01	1.33984E+01	1.86175E-01	6.98184E+00	1.13070E-01	1.38756E-12	2.67511E+01
F24	Avg	2.60000E+03	2.62755E+03	2.60563E+03	2.62739E+03	2.60000E+03	2.64230E+03	2.62596E+03	2.69070E+03
	Std	0.00000E+00	8.21927E+00	4.32802E+00	1.37930E+00	8.12379E-04	8.34590E+00	1.38743E+00	3.27067E+01
F25	Avg	**2.70000E+03**	2.71143E+03	2.71113E+03	2.70763E+03	2.71120E+03	2.71185E+03	2.70537E+03	2.71719E+03
	Std	**0.00000E+00**	5.52814E+00	1.56843E+01	1.40835E+00	4.56335E+00	3.58027E+00	1.23189E+00	9.81901E+00
F26	Avg	**2.70027E+03**	2.77377E+03	2.70380E+03	2.70045E+03	2.76350E+03	2.70050E+03	2.70034E+03	2.71116E+03
	Std	**7.15660E-02**	4.50143E+01	1.81704E+01	7.12784E-02	4.88044E+01	1.15199E-01	5.43145E-02	4.82765E+01
F27	Avg	**2.90000E+03**	3.41130E+03	3.69539E+03	3.10793E+03	3.34468E+03	3.37434E+03	3.10591E+03	3.61906E+03
	Std	**0.00000E+00**	2.99680E+02	4.03620E+02	2.43403E+00	1.11739E+02	1.47395E+02	2.10089E+00	1.98870E+02
F28	Avg	**3.00000E+03**	6.96685E+03	5.21016E+03	3.80003E+03	4.00390E+03	3.83997E+03	3.78391E+03	3.94223E+03
	Std	**0.00000E+00**	9.22570E+02	5.81678E+02	7.06540E+01	2.86352E+02	1.43014E+02	5.04540E+01	1.89334E+02
F29	Avg	**3.40123E+03**	4.54328E+04	5.73724E+06	3.93394E+03	7.37675E+05	2.57523E+06	3.90524E+03	3.20339E+06
	Std	**5.00388E+02**	9.87549E+04	4.68243E+06	9.58902E+01	1.51968E+06	4.79743E+06	7.66480E+01	3.65849E+06
F30	Avg	**3.86139E+03**	1.54413E+04	9.82272E+04	5.45281E+03	5.49253E+04	1.04715E+04	4.83092E+03	5.12597E+04
	Std	**2.38714E+02**	6.77786E+03	5.98133E+04	6.16567E+02	3.60247E+04	2.92969E+03	3.06931E+02	4.42759E+04

Function	Metric	HHO	BA	SCA	JAYA	TLBO	DE	SMA
F1	Avg	9.97423E+06	8.08199E+05	2.20166E+08	2.87552E+08	6.58450E+05	2.03311E+07	2.10690E+06
	Std	5.26966E+06	3.57865E+05	6.28888E+07	1.21955E+08	1.28854E+06	7.28222E+06	9.11719E+05
F2	Avg	1.20825E+07	6.36291E+05	1.62737E+10	4.85303E+10	2.04659E+02	6.38053E+02	6.43731E+03
	Std	2.82208E+06	3.38417E+05	2.39023E+09	1.15383E+10	7.91452E+00	1.91571E+03	1.02552E+04
F3	Avg	5.80049E+03	5.13058E+02	3.66024E+04	2.12622E+05	5.14536E+02	**3.81618E+02**	4.08070E+02
	Std	2.01773E+03	4.13993E+02	6.02024E+03	4.53268E+04	2.69510E+02	**7.62520E+01**	1.29899E+02
F4	Avg	5.42797E+02	4.33153E+02	1.47988E+03	1.13467E+04	4.77911E+02	4.98794E+02	4.96932E+02
	Std	4.14227E+01	3.95152E+01	2.85274E+02	4.65888E+03	3.81417E+01	2.22886E+01	4.15977E+01
F5	Avg	5.20248E+02	5.20950E+02	5.20934E+02	5.20908E+02	5.20950E+02	5.20570E+02	5.20806E+02
	Std	2.00569E-01	5.69218E-02	6.45071E-02	4.90918E-02	8.14438E-02	6.12389E-02	2.48426E-01
F6	Avg	6.30632E+02	6.32453E+02	6.33506E+02	6.38336E+02	6.18934E+02	6.19994E+02	6.14733E+02
	Std	3.00551E+00	3.94561E+00	2.28094E+00	3.36595E+00	2.88850E+00	1.62513E+00	3.27269E+00
F7	Avg	7.01115E+02	7.00584E+02	8.39379E+02	8.79197E+02	7.00096E+02	**7.00000E+02**	7.00287E+02
	Std	2.25070E-02	1.84311E-01	2.94778E+01	3.66214E+01	1.69074E-01	**5.98792E-11**	9.69376E-02
F8	Avg	8.95895E+02	1.02469E+03	1.04111E+03	1.10042E+03	8.90044E+02	8.00865E+02	8.12505E+02
	Std	1.76129E+01	5.43078E+01	2.17550E+01	4.21593E+01	1.38475E+01	9.83253E-01	3.49276E+00
F9	Avg	1.08627E+03	1.15936E+03	1.17420E+03	1.28023E+03	9.91781E+02	1.00810E+03	9.91688E+02
	Std	1.90858E+01	6.66046E+01	1.87304E+01	5.04828E+01	1.65109E+01	9.52898E+00	2.36080E+01
F10	Avg	2.86333E+03	5.31956E+03	6.80693E+03	4.54426E+03	3.08353E+03	1.02742E+03	1.63569E+03
	Std	6.07639E+02	6.98285E+02	5.08693E+02	1.83569E+03	5.92027E+02	3.00284E+01	1.98920E+02
F11	Avg	5.40578E+03	5.73029E+03	8.03029E+03	8.24579E+03	5.28450E+03	5.81604E+03	3.90039E+03
	Std	6.92539E+02	5.47433E+02	2.49962E+02	3.31757E+02	1.44160E+03	3.68120E+02	6.65242E+02
F12	Avg	1.20153E+03	1.20127E+03	1.20251E+03	1.20249E+03	1.20253E+03	1.20093E+03	1.20030E+03
	Std	5.30924E-01	4.57755E-01	2.90242E-01	2.51086E-01	2.49766E-01	1.34007E-01	1.45704E-01
F13	Avg	1.30048E+03	1.30048E+03	1.30298E+03	1.30612E+03	1.30047E+03	1.30034E+03	1.30052E+03
	Std	1.38590E-01	9.92765E-02	2.77158E-01	6.69230E-01	1.00015E-01	4.06942E-02	1.12004E-01
F14	Avg	1.40029E+03	1.40031E+03	1.44301E+03	1.47033E+03	1.40028E+03	1.40036E+03	1.40068E+03
	Std	1.51415E-01	1.06563E-01	8.00617E+00	1.72958E+01	6.03402E-02	8.83546E-02	2.94768E-01
F15	Avg	1.53793E+03	1.52819E+03	4.66572E+03	2.66362E+03	1.52844E+03	1.51168E+03	1.50620E+03
	Std	7.99928E+00	5.02099E+00	2.32154E+03	1.24713E+03	1.56136E+01	1.12207E+00	1.73461E+00
F16	Avg	1.61237E+03	1.61323E+03	1.61284E+03	1.61328E+03	1.61135E+03	1.61150E+03	1.61093E+03
	Std	3.28374E-01	4.17660E-01	2.41189E-01	1.72052E-01	4.82681E-01	2.73215E-01	6.44286E-01
F17	Avg	1.63637E+06	8.97661E+04	6.33771E+06	3.38028E+07	1.17429E+05	1.67523E+06	3.16965E+05
	Std	1.10939E+06	5.57390E+04	3.27333E+06	1.09951E+07	1.21736E+05	8.72545E+05	1.73061E+05
F18	Avg	9.35309E+04	9.45386E+04	1.55479E+08	8.35892E+08	5.45487E+03	7.55210E+03	2.08712E+04
	Std	4.04609E+04	3.71339E+04	7.51910E+07	5.72885E+08	5.29574E+03	4.49383E+03	7.46159E+03
F19	Avg	1.93473E+03	1.93673E+03	1.99079E+03	2.05412E+03	1.92194E+03	1.90834E+03	1.93177E+03
	Std	3.30943E+01	3.98173E+01	2.22810E+01	6.72141E+01	2.30056E+01	6.93852E-01	3.82442E+01
F20	Avg	1.46150E+04	2.46044E+03	1.77041E+04	9.50340E+04	2.54945E+03	4.80283E+03	2.21502E+03
	Std	6.85632E+03	1.68728E+02	4.99066E+03	7.69268E+04	1.97653E+02	1.65852E+03	2.12630E+02
F21	Avg	6.25701E+05	5.29042E+04	1.29594E+06	5.12880E+06	6.58189E+04	2.70672E+05	1.60505E+05
	Std	4.11998E+05	2.46847E+04	6.28852E+05	2.72402E+06	7.01267E+04	1.64876E+05	7.10942E+04
F22	Avg	3.04417E+03	3.36635E+03	2.97193E+03	3.55534E+03	2.54981E+03	**2.32993E+03**	2.69803E+03
	Std	2.92168E+02	3.23304E+02	1.81908E+02	1.86676E+02	1.35237E+02	**8.18740E+01**	2.23161E+02
F23	Avg	2.50000E+03	2.61525E+03	2.67168E+03	2.93275E+03	2.61524E+03	2.61524E+03	**2.50000E+03**
	Std	0.00000E+00	2.15818E-03	1.69671E+01	1.16873E+02	1.85747E-07	1.38756E-12	**0.00000E+00**
F24	Avg	2.60000E+03	2.66582E+03	2.60006E+03	2.64418E+03	2.60001E+03	2.62596E+03	2.60000E+03
	Std	5.63683E-05	2.06931E+01	4.34490E-02	6.92545E+01	7.30336E-04	2.91189E+00	0.00000E+00
F25	Avg	2.70000E+03	2.73390E+03	2.72551E+03	2.75887E+03	2.70000E+03	2.70734E+03	2.70000E+03
	Std	0.00000E+00	1.62934E+01	7.86095E+00	1.54773E+01	0.00000E+00	1.15088E+00	0.00000E+00
F26	Avg	2.76681E+03	2.70050E+03	2.70211E+03	2.71568E+03	2.72373E+03	2.70034E+03	2.70055E+03
	Std	4.77450E+01	1.35760E-01	6.81525E-01	5.53680E+01	4.27993E+01	2.89185E-02	1.63941E-01
F27	Avg	2.90000E+03	3.85363E+03	3.38213E+03	4.11693E+03	3.38111E+03	3.22890E+03	2.90000E+03
	Std	0.00000E+00	3.94149E+02	2.79554E+02	1.69233E+02	2.29824E+02	9.77594E+01	0.00000E+00
F28	Avg	3.00000E+03	5.49137E+03	4.81360E+03	4.94709E+03	4.13481E+03	3.64137E+03	3.00000E+03
	Std	0.00000E+00	8.74843E+02	3.57929E+02	3.06208E+02	3.72009E+02	1.96779E+01	0.00000E+00
F29	Avg	3.10000E+03	3.96585E+07	9.53550E+06	3.85900E+07	3.49051E+06	6.95627E+03	4.11959E+03
	Std	0.00000E+00	2.87107E+07	4.31435E+06	1.10169E+07	4.87224E+06	1.08154E+04	1.00655E+03
F30	Avg	5.55692E+03	1.91137E+04	2.09228E+05	5.69307E+05	7.37947E+03	6.36847E+03	5.48065E+03
	Std	5.74435E+03	3.96356E+04	7.94079E+04	3.12161E+05	4.18871E+03	1.19214E+03	2.13537E+03

**Table 6:** Average ranking values of involved conventional algorithms by Wilcoxon signed-rank test

Algorithm	Rank	+/ = /-	ARV
PSMADE	1	∼	2
PSO	10	28/1/1	9.133333
WOA	12	29/0/1	10.8
ABC	2	21/4/5	4.2
GWO	9	29/1/0	9.033333
SSA	6	29/1/0	6.733333
CS	3	24/5/4	4.4
MFO	13	30/0/0	11.46667
HHO	8	23/5/2	7.5
BA	11	29/0/1	9.566667
SCA	14	30/0/0	12.833333
JAYA	15	30/0/0	14.2
TLBO	7	27/2/1	6.833333
DE	5	25/0/5	5.866667
SMA	4	24/0/0	4.966667

**Figure 4:**
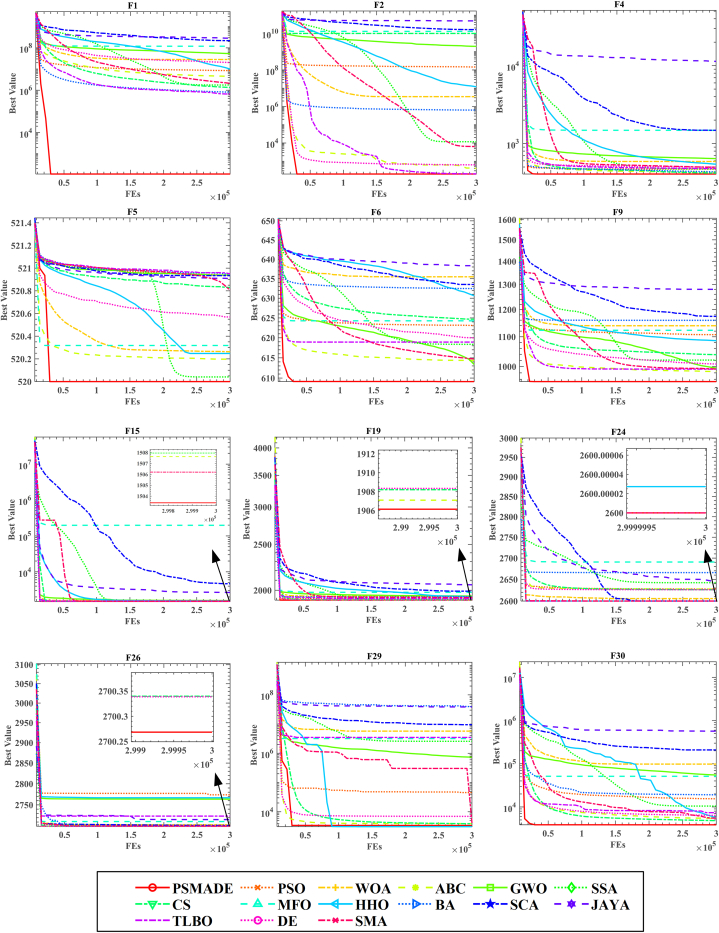
Fitness convergence comparison of PSMADE and 14 conventional MAs on CEC 2014 benchmark

To provide a more intuitive and clear demonstration of PSMADE’s convergence on the CEC 2014 benchmark, we have plotted its convergence curve alongside those of the 14 MAs participating in the comparison, as depicted in Figure 4. The resulting graph clearly illustrates that PSMADE outperforms all other algorithms in terms of both convergence speed and accuracy, thus solidifying its position as the undisputed champion over SMA.

Among unimodal functions, PSMADE ranks first on both F1 and F2, and second on F3 after CS. As shown in the subplots of Figure 4, PSMADE not only converges much faster than other algorithms compared but also exhibits a significant advantage in convergence accuracy. To further analyze this performance, we take F1 and F2 as examples. In F1, the average value for PSMADE is 1.09857E+02 while that for SMA is 2.10690E+06; in F2, the average value for PSMADE is 2.00000E+02 while that for SMA is 6.43731E+03. Based on the data presented above, it is evident that PSMADE exhibits superior performance in processing unimodal functions when compared to SMA. This can be attributed to incorporating the Powell mechanism, which facilitates deeper exploration of solution space domains and significantly enhances the algorithm’s ability to exploit local solution space domains.

As the multimodal function exhibits multiple peaks, employing it as a litmus test for evaluating the algorithm’s capacity to balance exploration and exploitation is more compelling. Based on Figure 4, it is evident that PSMADE outperforms its counterparts in terms of convergence accuracy when dealing with multimodal functions F4, F5, F6, F9, and F15. This is because adding the DE mechanism facilitates effective information exchange among excellent individuals, enabling the algorithm to obtain more sufficient candidate solutions within a superior population. It not only enhances the global exploration ability of the algorithm but also significantly improves its convergence accuracy.

Compared to the other three types of functions, PSMADE performs the worst on hybrid functions. Combining the data presented in Table 5, it becomes evident that PSMADE only attains first place in F19, while F17, F18, F20, and F21 are ranked second, all inferior to CS. It is placed fourth on F22, below DE, CS, and ABC. The experimental results demonstrate that the comprehensive performance of PSMADE on multimodal functions is second only to CS; furthermore, its convergence speed and accuracy have been considerably improved compared to SMA. Using the experimental results of F17 and F18 as an illustration, it can be observed that on F17, the mean value of PSMADE is 2.16208E+04 while the mean value of SMA is 3.16965E+05. Similarly, on F18, the average value of PSMADE is 1.87316E+03, with a corresponding average value of SMA being 2.08712E+04.

Finally, PSMADE performs commendably in processing composition functions and attains the optimal value and minimum standard deviation across eight composition functions F23-F30. Notably, F26, F29, and F30 demonstrate that PSMADE outperforms SMA in terms of convergence speed and accuracy. In situations where SMA is trapped in local optima leading to population search stagnation, PSMADE enables better escape from such states. The reason is that the DE mechanism enhances information exchange among dominant individuals, increases population diversity, mitigates the loss of diversity caused by super individuals, and significantly improves the algorithm’s global search capability. Moreover, the differential mutation operation incorporated in the DE mechanism endows the algorithm with the capability to evade local optima, thereby effectively preventing premature convergence. Simultaneously, the integration of Powell’s mechanism enhances the population’s local search ability, resulting in PSMADE exhibiting faster convergence speed and superior accuracy. The DE and Powell mechanisms complement each other, endowing PSMADE with excellent global exploration ability and strong local exploitation ability. This balance between exploration and exploitation ensures the optimal performance of PSMADE.

Based on the aforementioned indicators, PSMADE enhances the time disadvantage of original SMA when dealing with unimodal and multimodal problems while significantly augmenting the capability of original SMA to tackle various complex problems.

### Comparison with improved algorithms

In this section, we compare PSMADE with 15 improved advanced MAs on the CEC 2014 benchmark, including EBOwithCMAR,^80^ LSHADE_cnEpSi,^81^ JADE,^82^ HHODE,^83^ ALCPSO,^84^ CLPSO,^85^ LSHADE,^86^ SADE,^87^ SHADE,^88^ RCBA,^89^ CBA,^90^ LWOA,^91^ IWOA,^92^ IGWO,^93^ LGWO.^94^ The parameter settings for all the improved algorithms involved in this experiment are listed in Table 7.

**Table 7:** Parameter setting of the comparison improved algorithms with PSMADE

Algorithm	Other parameters
PSMADE	z=0.03;ScalingFactor=[0.4,1];CrossoverProbability=[0.5,1]
EBOwithCMAR	$ce=0;PS_{1}=30;PS_{2}=14;PS=44$
LSHADE_cnEpSi	$freq_{init}=0.5;arc=1.4;p=0.11;pb=0.4;ps=0.5;memory\_size=5$
JADE	Afactor=1;p=0.05;c=0.1;CRm=0.5;Fm=0.5
HHODE	$c=\left[2,0\right];Escaping\_Energy=\left[-c,c\right];P_{CR}=0.9;beta=0.5$
ALCPSO	$lifespan=60;c_{1}=c_{2}=2;T=2;w=0.4;pro=\frac{1}{N}$
CLPSO	c=1.496;w∈[0.9,0.2];m=5
LSHADE	p=0.1;arc=2;ms=5
SADE	L=50
SHADE	P=0.1;arc=2
RCBA	$r=ones\left(1,N\right)*0.5;f_{\min}=0;f_{\max}=2$
CBA	$r1=r2=0.5+rand\left(0,1\right);f\;\max=2.5;Cw=3;\;\omega =\left(\frac{FEs}{MaxFEs}\right)+0.1;u=\left(\frac{FEs}{MaxFEs}\right)*0.4+0.3$
LWOA	a=[2,0];b=1;a2=[−1,−2];beta=1.5
IWOA	a=[2,0];b=1;a2=[−1,−2];CR=0.1
IGWO	a=[2,0]
LGWO	a=[2,0];q=2

The experimental results of PSMADE compared with 15 improved algorithms on the CEC 2014 benchmark are given in Table 8. Analysis of the data in Table 8 shows that PSMADE obtained optimal values of Avg and Stdv values measuring the quality of the solution and the stability of the algorithm, respectively, on 12 CEC 2014 benchmarks, including F1, F5, F18, F20, F23, F24, F25, F26, F27, F28, F29, and F30. PSMADE is the winner in terms of convergence accuracy and convergence speed on the unimodal benchmark functions F1, F2, and F3, the multimodal function F5, the hybrid functions F18 and F20, and the composition functions F23-F30.

**Table 8:** Results of PSMADE and improved algorithms on CEC 2014 benchmark

Function	Metric	PSMADE	EBOwithCMAR	LSHADE_cnEpSi	JADE	HHODE	ALCPSO	CLPSO	LSHADE
F1	Avg	**1.12097E+02**	4.33078E+03	5.75943E+03	1.19864E+03	1.08565E+07	3.76568E+06	6.03749E+06	8.68517E+03
	Std	**4.26006E+01**	6.00577E+03	3.39937E+03	2.25657E+03	5.28222E+06	4.24727E+06	1.98906E+06	1.24464E+04
F2	Avg	2.00000E+02	**2.00000E+02**	2.00000E+02	2.00000E+02	1.45634E+07	2.15253E+03	5.79577E+02	2.00000E+02
	Std	7.24483E-07	**1.97476E-14**	2.23904E-09	1.31839E-13	4.23080E+06	3.71287E+03	1.39691E+03	1.51133E-13
F3	Avg	3.00000E+02	3.00576E+02	3.00000E+02	3.00000E+02	4.12854E+03	4.66167E+02	4.58340E+02	3.00000E+02
	Std	1.02126E-08	2.18782E+00	**2.08212E-12**	1.13687E-13	2.37382E+03	4.79983E+02	1.84987E+02	1.03265E-11
F4	Avg	4.02617E+02	4.00476E+02	4.10685E+02	**4.00000E+02**	5.45616E+02	5.14593E+02	4.63431E+02	4.08628E+02
	Std	4.80146E+00	1.23621E+00	2.78315E+01	**4.67893E-06**	3.73282E+01	4.39541E+01	2.25229E+01	2.18630E+01
F5	Avg	**5.20000E+02**	5.20001E+02	5.20011E+02	5.20043E+02	5.20390E+02	5.20803E+02	5.20313E+02	5.20005E+02
	Std	**5.07697E-05**	1.77395E-03	5.53928E-03	1.38327E-02	1.06022E-01	5.38705E-02	4.12383E-02	1.45559E-02
F6	Avg	6.09568E+02	6.08532E+02	6.11868E+02	**6.06161E+02**	6.19459E+02	6.16165E+02	6.12244E+02	6.10837E+02
	Std	3.00410E+00	3.09392E+00	2.51668E+00	**3.28856E+00**	3.30748E+00	3.37199E+00	1.32702E+00	2.31851E+00
F7	Avg	7.00004E+02	**7.00000E+02**	7.00022E+02	7.00006E+02	7.01166E+02	7.00020E+02	7.00000E+02	7.00014E+02
	Std	6.70911E-03	**5.17115E-14**	2.41975E-02	8.41605E-03	4.86053E-02	2.53402E-02	4.70840E-06	1.78230E-02
F8	Avg	8.03648E+02	8.01492E+02	8.03018E+02	**8.00000E+02**	8.77772E+02	8.24039E+02	8.00000E+02	8.00033E+02
	Std	1.94363E+00	1.86140E+00	3.16238E+00	**0.00000E+00**	2.24057E+01	9.89337E+00	1.13687E-13	1.81654E-01
F9	Avg	9.49914E+02	**9.23968E+02**	9.41872E+02	9.32723E+02	1.02523E+03	1.01747E+03	9.52791E+02	9.34028E+02
	Std	1.22446E+01	**6.09422E+00**	9.03265E+00	7.29902E+00	3.17609E+01	3.15784E+01	7.13686E+00	9.33500E+00
F10	Avg	1.18280E+03	1.04798E+03	1.08889E+03	1.01226E+03	2.42065E+03	1.59886E+03	**1.00021E+03**	1.00797E+03
	Std	1.28828E+02	7.36256E+01	8.79818E+01	3.61050E+01	4.85462E+02	2.63635E+02	**3.33284E-01**	3.00573E+01
F11	Avg	3.13864E+03	2.79186E+03	**2.76001E+03**	2.84372E+03	4.56307E+03	4.45060E+03	3.14068E+03	2.86645E+03
	Std	4.66519E+02	2.66470E+02	**3.07111E+02**	3.06787E+02	9.46492E+02	6.86588E+02	3.17661E+02	3.58692E+02
F12	Avg	1.20032E+03	1.20018E+03	1.20011E+03	1.20012E+03	1.20119E+03	1.20139E+03	1.20033E+03	1.20013E+03
	Std	1.34756E-01	3.89110E-02	1.87919E-02	2.16837E-02	6.80804E-01	5.10038E-01	5.55728E-02	1.25390E-01
F13	Avg	1.30030E+03	**1.30027E+03**	1.30034E+03	1.30028E+03	1.30043E+03	1.30054E+03	1.30029E+03	1.30031E+03
	Std	5.87244E-02	**7.74232E-02**	9.15365E-02	8.63082E-02	1.17191E-01	7.67734E-02	4.97937E-02	5.39541E-02
F14	Avg	1.40044E+03	1.40028E+03	1.40032E+03	1.40027E+03	1.40026E+03	1.40051E+03	1.40027E+03	1.40034E+03
	Std	2.39928E-01	5.75133E-02	1.16234E-01	5.23318E-02	5.96465E-02	2.82040E-01	2.79913E-02	1.55609E-01
F15	Avg	1.50333E+03	1.50386E+03	1.50735E+03	1.50318E+03	1.52181E+03	1.51192E+03	1.50731E+03	1.50481E+03
	Std	1.01505E+00	1.03680E+00	5.17952E+00	8.00554E-01	1.18091E+01	5.01739E+00	1.37616E+00	1.31297E+00
F16	Avg	1.61029E+03	1.60922E+03	**1.60915E+03**	1.60959E+03	1.61168E+03	1.61181E+03	1.60990E+03	1.60947E+03
	Std	7.43831E-01	5.89364E-01	**4.21038E-01**	3.71230E-01	4.41231E-01	3.66188E-01	4.73564E-01	7.35598E-01
F17	Avg	2.48210E+04	5.65308E+03	3.62563E+03	4.13897E+04	1.52761E+06	5.99996E+05	8.38006E+05	**3.15714E+03**
	Std	2.18452E+04	5.81566E+03	5.05437E+02	1.21951E+05	9.85682E+05	6.59849E+05	4.27565E+05	**4.06979E+02**
F18	Avg	**1.88863E+03**	1.90055E+03	1.93277E+03	2.23038E+03	5.82880E+03	7.69809E+03	1.89697E+03	1.95141E+03
	Std	**1.48810E+02**	3.23131E+01	4.51105E+01	1.46164E+03	4.86297E+03	7.05835E+03	7.22539E+01	4.83280E+01
F19	Avg	1.90603E+03	1.90869E+03	1.91222E+03	1.90851E+03	1.92047E+03	1.91390E+03	1.90735E+03	1.91404E+03
	Std	1.97176E+00	1.81512E+00	1.03843E+01	1.24956E+01	1.93242E+01	1.59612E+01	6.05300E-01	1.98452E+01
F20	Avg	**2.13010E+03**	2.97137E+03	2.29764E+03	6.06817E+03	5.57827E+03	2.96242E+03	4.94993E+03	2.18058E+03
	Std	**6.85768E+01**	2.95692E+03	1.17746E+02	4.99891E+03	2.40852E+03	5.36128E+02	1.83541E+03	7.57621E+01
F21	Avg	1.56693E+04	3.25710E+03	3.03260E+03	3.01504E+04	3.78068E+05	8.48987E+04	8.93065E+04	**2.74953E+03**
	Std	1.39007E+04	3.34315E+02	2.76526E+02	8.32709E+04	2.64961E+05	9.01191E+04	5.08263E+04	**2.46526E+02**
F22	Avg	2.47532E+03	2.45242E+03	2.44424E+03	2.42289E+03	2.66481E+03	2.57369E+03	2.40542E+03	2.44091E+03
	Std	1.88719E+02	1.09572E+02	8.93908E+01	9.19923E+01	1.78217E+02	1.51607E+02	9.64851E+01	1.15204E+02
F23	Avg	**2.50000E+03**	2.61524E+03	2.61494E+03	2.61524E+03	2.50000E+03	2.61530E+03	2.61524E+03	2.61524E+03
	Std	**0.00000E+00**	3.59280E-06	1.45854E-01	1.70987E-12	0.00000E+00	2.39898E-01	2.77191E-06	2.04418E-12
F24	Avg	**2.60000E+03**	2.62285E+03	2.64082E+03	2.63463E+03	2.60000E+03	2.63595E+03	2.62512E+03	2.64280E+03
	Std	**0.00000E+00**	8.11387E+00	6.93872E+00	6.58750E+00	3.13644E-04	6.36424E+00	6.58975E-01	6.16819E+00
F25	Avg	**2.70000E+03**	2.70645E+03	2.70881E+03	2.70781E+03	2.70000E+03	2.71081E+03	2.70743E+03	2.70540E+03
	Std	**0.00000E+00**	3.05998E+00	4.16706E+00	2.29859E+00	0.00000E+00	3.55543E+00	8.16427E-01	3.16362E+00
F26	Avg	**2.70028E+03**	2.71028E+03	2.72365E+03	2.70703E+03	2.70045E+03	2.76529E+03	2.70037E+03	2.71362E+03
	Std	6.45662E-02	3.04211E+01	4.28517E+01	2.52738E+01	1.50494E-01	7.87213E+01	6.92246E-02	3.44699E+01
F27	Avg	**2.90000E+03**	3.17381E+03	3.29712E+03	3.13882E+03	2.90000E+03	3.42285E+03	3.11354E+03	3.28204E+03
	Std	**0.00000E+00**	7.85417E+01	1.41031E+02	6.57608E+01	0.00000E+00	2.31279E+02	8.02802E+00	1.00678E+02
F28	Avg	**3.00000E+03**	3.85992E+03	3.97132E+03	3.67211E+03	3.00000E+03	4.37844E+03	3.71968E+03	3.77099E+03
	Std	**0.00000E+00**	1.40829E+02	1.98944E+02	1.16669E+02	0.00000E+00	4.52193E+02	7.11100E+01	1.36562E+02
F29	Avg	**3.40497E+03**	3.01595E+05	3.67608E+03	3.92713E+05	3.10434E+05	2.42229E+06	3.82254E+03	3.15116E+05
	Std	**4.36641E+02**	1.62738E+06	6.41565E+01	2.13055E+06	1.67260E+06	6.35965E+06	1.24801E+02	1.70585E+06
F30	Avg	**3.89017E+03**	6.33677E+03	5.70787E+03	5.57781E+03	1.65134E+04	1.35396E+04	6.25220E+03	5.58622E+03
	Std	**2.51064E+02**	4.24407E+03	1.27114E+03	1.17774E+03	1.45589E+04	9.10207E+03	7.52121E+02	9.55020E+02

Function	Metric	SADE	SHADE	RCBA	CBA	LWOA	IWOA	IGWO	LGWO
F1	Avg	4.41625E+05	2.18812E+03	1.20045E+06	4.13849E+06	4.05268E+06	2.08040E+07	1.65192E+07	3.74720E+08
	Std	2.80151E+05	3.19251E+03	4.46889E+05	1.69333E+06	1.39712E+06	1.31502E+07	6.12272E+06	5.66707E+07
F2	Avg	2.00000E+02	2.00000E+02	2.85920E+04	1.15884E+04	5.27082E+05	6.85370E+06	3.06703E+06	2.34790E+10
	Std	1.78835E-08	2.84217E-14	1.10279E+04	8.58185E+03	1.53157E+05	1.92822E+07	1.59796E+06	2.18125E+09
F3	Avg	4.69344E+02	3.00000E+02	3.24875E+02	5.99103E+03	9.02101E+02	2.04229E+04	6.63488E+03	4.66694E+04
	Std	5.71185E+02	2.10847E-13	8.77306E+00	6.84519E+03	2.98744E+02	1.32829E+04	2.18574E+03	5.69716E+03
F4	Avg	4.40074E+02	4.06794E+02	4.79874E+02	5.07446E+02	5.08565E+02	5.60133E+02	5.24922E+02	2.47205E+03
	Std	4.35548E+01	1.94080E+01	4.10133E+01	3.53639E+01	3.64575E+01	3.56889E+01	2.68376E+01	4.62698E+02
F5	Avg	5.20516E+02	5.20011E+02	5.20059E+02	5.20158E+02	5.20451E+02	5.20255E+02	5.20475E+02	5.20947E+02
	Std	4.27023E-02	2.56782E-03	6.44028E-02	1.87180E-01	1.25638E-01	1.61274E-01	1.00081E-01	5.07290E-02
F6	Avg	6.09040E+02	6.07815E+02	6.38066E+02	6.40464E+02	6.29465E+02	6.30586E+02	6.18978E+02	6.36283E+02
	Std	1.89627E+00	3.25098E+00	2.95702E+00	2.79612E+00	3.48614E+00	4.09053E+00	2.99172E+00	1.60386E+00
F7	Avg	7.00020E+02	7.00007E+02	7.00067E+02	7.00023E+02	7.00672E+02	7.00905E+02	7.00989E+02	9.28124E+02
	Std	3.73518E-02	1.16692E-02	2.12456E-02	5.69988E-02	1.05290E-01	1.37294E-01	4.81903E-02	2.31714E+01
F8	Avg	8.01658E+02	8.00133E+02	1.01183E+03	1.02331E+03	8.68788E+02	9.18685E+02	8.84295E+02	1.06116E+03
	Std	1.17822E+00	7.26614E-01	5.61642E+01	4.35938E+01	1.65707E+01	1.87836E+01	1.72735E+01	1.60039E+01
F9	Avg	9.42054E+02	9.30316E+02	1.15540E+03	1.14185E+03	1.12484E+03	1.10997E+03	1.01066E+03	1.17817E+03
	Std	1.09988E+01	5.88835E+00	6.32420E+01	4.77074E+01	6.27572E+01	3.78939E+01	2.07817E+01	1.25746E+01
F10	Avg	1.00573E+03	1.00842E+03	5.74856E+03	5.82463E+03	2.04641E+03	2.77162E+03	3.39457E+03	7.90023E+03
	Std	2.16329E+01	2.99517E+01	8.10965E+02	7.50261E+02	3.46940E+02	4.65773E+02	5.62751E+02	3.40055E+02
F11	Avg	2.98855E+03	2.85327E+03	5.66966E+03	5.83827E+03	5.27719E+03	5.30451E+03	4.49091E+03	8.15377E+03
	Std	4.24962E+02	3.39268E+02	7.15629E+02	5.36995E+02	6.03178E+02	8.48363E+02	7.68050E+02	3.74930E+02
F12	Avg	1.20070E+03	**1.20011E+03**	1.20053E+03	1.20114E+03	1.20085E+03	1.20100E+03	1.20070E+03	1.20245E+03
	Std	7.66967E-02	**2.46500E-02**	2.34233E-01	3.49681E-01	3.09211E-01	3.69568E-01	3.11974E-01	2.92009E-01
F13	Avg	1.30028E+03	1.30031E+03	1.30049E+03	1.30051E+03	1.30055E+03	1.30053E+03	1.30058E+03	1.30398E+03
	Std	5.07710E-02	8.28648E-02	1.13998E-01	1.26768E-01	1.13424E-01	1.12825E-01	1.15690E-01	2.47031E-01
F14	Avg	**1.40024E+03**	1.40033E+03	1.40029E+03	1.40037E+03	1.40028E+03	1.40027E+03	1.40038E+03	1.47759E+03
	Std	**3.66347E-02**	1.36751E-01	7.01820E-02	1.69745E-01	4.33932E-02	4.92272E-02	2.98360E-01	8.53620E+00
F15	Avg	1.50647E+03	**1.50313E+03**	1.53702E+03	1.56057E+03	1.52106E+03	1.56018E+03	1.51551E+03	1.27012E+04
	Std	2.30052E+00	**6.79152E-01**	9.55210E+00	1.69508E+01	4.89660E+00	1.99481E+01	3.59558E+00	4.93603E+03
F16	Avg	1.61055E+03	1.60935E+03	1.61344E+03	1.61339E+03	1.61265E+03	1.61253E+03	1.61172E+03	1.61285E+03
	Std	4.84808E-01	7.03814E-01	4.02703E-01	3.10418E-01	3.72153E-01	5.61621E-01	6.84455E-01	2.52363E-01
F17	Avg	5.39208E+04	3.16883E+03	1.29011E+05	2.82893E+05	6.40334E+05	3.09158E+06	9.26169E+05	8.03248E+06
	Std	4.22166E+04	3.81606E+02	9.77292E+04	1.57262E+05	2.83522E+05	1.79642E+06	5.72456E+05	3.41898E+06
F18	Avg	2.71655E+03	1.94507E+03	1.11717E+04	9.87050E+03	1.05503E+04	1.09021E+04	2.41245E+04	2.05557E+08
	Std	1.40819E+03	5.69635E+01	1.09042E+04	8.40388E+03	1.23579E+04	2.21157E+04	3.51308E+04	9.27845E+07
F19	Avg	1.90674E+03	1.90579E+03	1.92464E+03	1.92999E+03	1.92727E+03	1.94347E+03	1.92085E+03	2.01667E+03
	Std	1.08752E+01	**1.06905E+00**	2.58107E+01	2.53723E+01	3.23692E+01	4.48066E+01	1.84465E+01	1.90301E+01
F20	Avg	3.12645E+03	2.17751E+03	2.40982E+03	2.93412E+03	3.03369E+03	1.57407E+04	3.17035E+03	2.36329E+04
	Std	1.20063E+03	7.47546E+01	1.39629E+02	7.56893E+02	6.41313E+02	9.38441E+03	9.27083E+02	8.02891E+03
F21	Avg	2.07046E+04	2.75547E+03	7.87072E+04	9.47816E+04	2.16591E+05	8.18429E+05	3.21466E+05	2.37071E+06
	Std	1.89649E+04	2.32155E+02	4.88862E+04	4.67006E+04	2.06629E+05	6.81499E+05	2.92593E+05	8.96005E+05
F22	Avg	**2.40439E+03**	2.43175E+03	3.36714E+03	3.39359E+03	2.90019E+03	2.84405E+03	2.59662E+03	3.23052E+03
	Std	**9.72631E+01**	1.23286E+02	4.22618E+02	3.75556E+02	2.11052E+02	1.69063E+02	1.56777E+02	1.55392E+02
F23	Avg	2.61524E+03	2.61524E+03	2.61525E+03	2.61578E+03	2.61542E+03	2.61981E+03	2.62137E+03	2.72030E+03
	Std	1.33518E-12	1.56165E-12	4.26698E-03	2.35254E-01	9.01591E-02	2.09472E+00	3.12716E+00	2.30333E+01
F24	Avg	2.63172E+03	2.63409E+03	2.67252E+03	2.67515E+03	2.60444E+03	2.60261E+03	2.60001E+03	2.60000E+03
	Std	4.99888E+00	6.97270E+00	2.61344E+01	2.70422E+01	5.54186E+00	1.76688E+00	4.09949E-03	4.86976E-09
F25	Avg	2.71090E+03	2.70493E+03	2.73140E+03	2.73293E+03	2.71885E+03	2.71553E+03	2.71040E+03	2.70000E+03
	Std	2.18201E+00	1.97306E+00	1.68219E+01	2.03315E+01	1.04665E+01	1.32286E+01	2.38993E+00	1.46262E-13
F26	Avg	2.71689E+03	2.70371E+03	2.71710E+03	2.70382E+03	2.70048E+03	2.70374E+03	2.70074E+03	2.70330E+03
	Std	3.78166E+01	1.81900E+01	3.77753E+01	1.82564E+01	1.04485E-01	1.81813E+01	1.77235E-01	2.40891E-01
F27	Avg	3.17902E+03	3.18694E+03	3.88180E+03	4.01990E+03	3.65205E+03	3.61347E+03	3.11040E+03	3.44727E+03
	Std	7.65075E+01	8.62136E+01	4.88491E+02	4.22291E+02	3.44449E+02	3.35484E+02	3.81213E+00	2.49340E+02
F28	Avg	3.71995E+03	3.72157E+03	5.78038E+03	5.33833E+03	4.53855E+03	4.54990E+03	3.82833E+03	5.47109E+03
	Std	3.49325E+01	9.73647E+01	1.23292E+03	6.78767E+02	3.70275E+02	5.20391E+02	1.44019E+02	2.06620E+02
F29	Avg	4.01888E+03	3.94449E+05	1.30666E+07	3.96821E+07	6.71592E+06	6.31911E+06	1.87950E+06	2.20942E+07
	Std	2.06796E+02	2.14040E+06	1.59102E+07	4.36528E+07	3.76641E+06	4.59685E+06	4.25118E+06	8.85299E+06
F30	Avg	5.26737E+03	5.55292E+03	1.86083E+04	3.44395E+04	1.33490E+04	2.61403E+04	2.79065E+04	4.18805E+05
	Std	6.28726E+02	1.10322E+03	3.14675E+04	7.26489E+04	1.43735E+04	2.10759E+04	9.57433E+03	1.18249E+05

This is attributed to implementing the DE mechanism, which incorporates a supplementary population consisting of exceptional individuals to update the current position. This facilitates closer communication among individuals and effectively harnesses information from outstanding members. Meanwhile, the crossover and mutation operations within the DE mechanism augment population diversity, enabling the algorithm to escape local optima in cases of premature convergence and achieve superior global exploration performance. Furthermore, the incorporation of Powell’s method allows for a more thorough exploration of the solution space, enabling individuals within the population to fully utilize feasible solutions nearby and ultimately enhancing the convergence accuracy of the algorithm.

**Figure 5:**
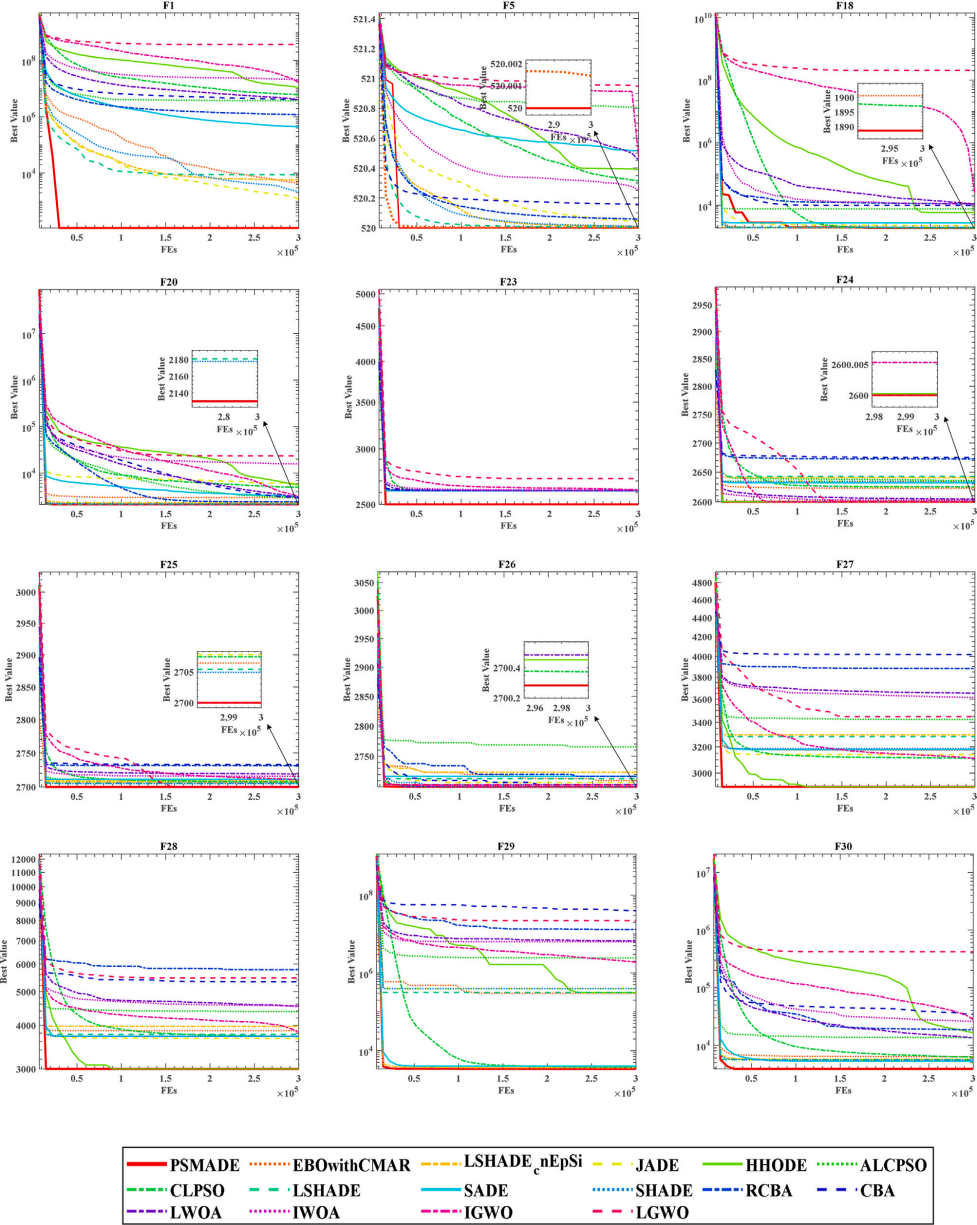
Fitness convergence comparison of PSMADE and 15 improved MAs on CEC 2014 benchmark

In addition, to show more intuitively the results of PSMADE and the 15 improved algorithms involved in the comparison on the CEC 2014 benchmark, we plotted the convergence curves of all the above algorithms, as depicted in Figure 5. In addition, we also identified the other algorithms for which PSMADE outperformed, equaled, or underperformed the CEC 2014 benchmark by the symbol "+/ = /-", respectively, and the specific results and the average ranking results of each algorithm are recorded in Table 9. As per the final results, PSMADE has an AVR of 4, ranking first among the 16 algorithms involved in the comparison.

**Table 9:** Average ranking values of involved improved algorithms by Wilcoxon signed-rank test

Algorithm	Rank	+/ = /-	ARV
**PSMADE**	**1**	**∼**	**4**
EBOwithCMAR	4	12/6/12	4.8
LSHADE_cnEpSi	6	17/4/9	6.1
JADE	3	14/5/11	4.7
HHODE	9	24/5/1	9.3
ALCPSO	10	29/1/0	10.533333
CLPSO	7	19/7/4	6.3
LSHADE	5	15/5/10	5.566667
SADE	8	18/7/5	6.466667
SHADE	2	12/7/11	4.266667
RCBA	13	29/0/1	11.766667
CBA	15	29/1/0	12.833333
LWOA	11	29/0/1	11.133333
IWOA	14	29/0/1	12.466667
IGWO	11	29/1/0	11.133333
LGWO	16	28/2/0	14.366667

In conclusion, incorporating complementary DE and Powell mechanisms in PSMADE facilitates a more optimal balance between global exploration and local exploitation, thereby endowing it with robustness and versatility to tackle diverse complex optimization problems.

### Experiments on engineering benchmarks

As it is known that practice is the only test of truth, in this section, we will use the proposed PSMADE algorithm to solve realistic constrained practical engineering problems, in reality, namely, tension/compression spring design problem (TCSD), the Belleville spring design problem (BSDP), the hydrostatic thrust bearing problem (HTBP), and the cantilever structure problem (SCP). The experiment in this section further proves the feasibility and superiority of PSMADE by comparing the ability of PSMADE and other algorithms to solve the same practical engineering problem.

### Tension/compression spring design problem

This experiment aims to minimize the mass of the tension/compression spring while meeting all constraints professionally. The weight of the tension/compression spring depends on three key factors the wire diameter, the average coil diameter, and the number of active coils. Therefore, in order to achieve the objectives of this experiment, the following mathematical model was developed, which includes three design variables, namely the wire diameter (d), the average coil diameter (D), and the number of active coils (N). The mathematical model describing the problem is as follows.

$$Consider:\vec{x}=\left[x_{1},x_{2},x_{3}\right]=\left[d,D,N\right]$$

$$Object:F{\left(\vec{x}\right)}_{\min}=x_{1}^{2}\cdot x_{2}\cdot x_{3}+2x_{1}^{2}\cdot x_{2}$$

$$Subject\;to:H_{1}\left(\vec{x}\right)=1-\frac{x_{2}^{3}\cdot x_{3}}{71785x_{1}^{4}}\leq 0$$

$$H_{2}\left(\vec{x}\right)=\frac{4x_{2}^{2}-x_{1}\cdot x_{2}}{12566\left(x_{2}\cdot x_{1}^{3}-x_{1}^{4}\right)}+\frac{1}{5180x_{1}^{2}}-1\leq 0$$

$$H_{3}\left(\vec{x}\right)=\frac{x_{1}+x_{2}}{1.5}-1\leq 0$$

$$H_{4}\left(\vec{x}\right)=1-\frac{140.45x_{1}}{x_{2}^{3}\cdot x_{3}}\leq 0$$

**Variable ranges**: $0.05\leq x_{1}\leq 2.00$, $0.25\leq x_{2}\leq 1.30$, $2.00\leq x_{3}\leq 15.00$

Table 10 displays the results of PSMADE with GA,^95^ WOA,^72^ GWO,^73^ SCADE,^96^ and mathematical and constraint methods in solving this problem. According to the table, PSMADE obtains a solution of 0.012665233, the best result among all the above methods. Therefore, it can be concluded that PSMADE on TCSD has better results and some improvement results.

**Table 10:** Results for each algorithm in the tension/compression spring experiment

Algorithm	*D*	*D*	*N*	Best Cost
PSMADE	0.051687035	0.356669002	11.29182369	0.012665233
GA	0.051480	0.351661	11.632201	0.0127048
WOA	0.051207	12.0043032	0.345215	0.0126763
GWO	0.055172952	0.44276283	7.681401301	0.013048537
SCADE	0.05	0.31447915	15	0.013365364
Constraint correction	0.053396	901854000	0.399180	0.0127303
Mathematical Optimization	0.050000	14.250000	0.315900	0.0128334

### Belleville spring design problem

The ultimate goal of this experiment is to minimize the weight of the Belleville spring under the premise of meeting multiple constraints. The weight of the spring depends on the following four design variables, namely, the spring outer diameter (De), the spring inner diameter (Di), the spring thickness (t), and the spring height (h). According to these four variables, the spring quality problem is modeled, and the mathematical model is as follows.

$$Consider:\vec{x}=\left[x_{1},x_{2},x_{3},x_{4}\right]=\left[De,Di,t,h\right]$$

$$Object:F{\left(\vec{x}\right)}_{\min}=0.07075\pi \left(x_{1}^{2}-x_{2}^{2}\right)\cdot x_{3}$$

$$Subject\;to:H_{1}\left(\vec{x}\right)=S-\frac{4E\delta_{\max}}{\left(1-\mu^{2}\right)\alpha x_{1}^{2}}\left[\beta \left(x_{4}-\frac{\delta_{\max}}{2}\right)+\gamma x_{3}\right]\geq 0$$

$$H_{2}\left(\vec{x}\right)=\frac{4E\delta_{\max}}{\left(1-\mu^{2}\right)\alpha x_{1}^{2}}\left[\left(x_{4}-\frac{\delta_{\max}}{2}\right)\left(x_{4}-\delta_{\max}\right)x_{3}+x_{3}^{3}\right]-P_{\max}\geq 0$$

$$H_{3}\left(\vec{x}\right)=\delta_{1}-\delta_{\max}\geq 0$$

$$H_{4}\left(\vec{x}\right)=H-x_{3}-x_{4}\geq 0$$

$$H_{5}\left(\vec{x}\right)=D_{\max}-x_{1}\geq 0$$

$$H_{6}\left(\vec{x}\right)=x_{1}-x_{2}\geq 0$$

$$H_{7}\left(\vec{x}\right)=0.3-\frac{x_{4}}{x_{1}-x_{2}}\geq 0$$

**Variable ranges**: $1\leq R,R_{0},Q\leq 16$, $K=\frac{x_{1}}{x_{2}}$, 1e−6≤μ≤16e−6, $P_{\max}=5400$,

$$\alpha =\frac{6}{\pi \;\ln\;K}{\left(\frac{K-1}{K}\right)}^{2},\beta =\frac{6}{\pi \;\ln\;K}\left(\frac{K-1}{\ln\;K}-1\right),\gamma =\frac{6}{\pi \;\ln\;K}\left(\frac{K-1}{2}\right),E=30e6psi,\mu =0.3,\delta_{\max}=0.2,S=200KPsi,D_{\max}=12.01,H=2,\delta_{1}=f\left(a\right)x_{4},a=\frac{x_{4}}{x_{3}}\text{,}$$

the defined values of f(a) are shown in Table 11.

**Table 11:** Values of a and f(a)

a	≤1.4	1.5	1.6	1.7	1.8	1.9	2.0	2.1	2.2	2.3	2.4	2.5	2.6	2.7	≥2.8
f(a)	1	0.85	0.77	0.71	0.66	0.63	0.6	0.58	0.56	0.55	0.53	0.52	0.51	0.51	0.50

The results of PSMADE with NDE,^97^ MBA,^98^ TLBO,^99^ Gene AS I,^100^ and Gene AS II^100^ in solving this problem are recorded in Table 12. We can see that PSMADE obtains a solution of 1.979674757, the best result among all the above methods.

**Table 12:** Results for each algorithm in the belleville spring design experiment

Algorithm	*d*	*D*	*N*	Best Cost
PSMADE	12.01	10.03047329	0.204143354	0.2
NDE	12.009999	10.030473	0.204143	0.200000
MBA	12.010000	10.030473	0.204143	0.200000
TLBO	12.010000	10.030473	0.204143	0.200000
Gene AS I	11.627000	9.354000	0.205000	0.201000
Gene AS II	11.499000	9.268000	0.210000	0.204000

### Hydrostatic thrust bearing problem

The aim of this experiment is to minimize the power dissipation of the stationary thrust bearing during its operation. Since this bearing must be subjected to a certain load while providing axial support, this experiment must also satisfy a series of constraints. There are four design variables for this experiment: bearing step radius (R), groove radius ($R_{0}$), oil viscosity (μ), and flow rate (Q). The mathematical model of the problem is as follows.

$$Consider:\vec{x}=\left[x_{1},x_{2},x_{3},x_{4}\right]=\left[R,R_{0},\mu ,Q\right]$$

$$Object:F{\left(\vec{x}\right)}_{\min}=\frac{QP_{0}}{0.7}+E_{f}$$

$$Subject\;to:H_{1}\left(\vec{x}\right)=\frac{\pi P_{0}}{2}\cdot \frac{x_{1}^{2}-x_{2}^{2}}{\ln\left(\frac{x_{1}}{x_{2}}\right)}-W_{s}\geq 0$$

$$H_{2}\left(\vec{x}\right)=P_{\max}-P_{0}\geq 0$$

$$H_{3}\left(\vec{x}\right)=\Delta T_{\max}-\Delta T\geq 0$$

$$H_{4}\left(\vec{x}\right)=5000-\frac{w}{\pi \left(x_{1}^{2}-x_{2}^{2}\right)}\geq 0$$

$$H_{5}\left(\vec{x}\right)=R-R_{0}\geq 0$$

$$H_{6}\left(\vec{x}\right)=h-h_{\min}\geq 0$$

$$H_{7}\left(\vec{x}\right)=0.001-\frac{\gamma }{gP_{0}\left(\frac{x_{4}}{2\pi Rh}\right)}\geq 0$$

**Variable ranges**: $P_{0}=\frac{6x_{3}\cdot x_{4}}{\pi h^{3}}\ln\left(\frac{x_{1}}{x_{2}}\right)$, $\Delta T=2\left(10^{P}-560\right)$, $E_{f}=9336\lambda C\Delta Tx_{4}$,

$$P=\frac{\log\left(\log\left(8.122\times 10^{6}+0.8\right)\right)-C_{1}}{n},C_{1}=10.04,C_{1}=10.04,N=750\text{,}$$

$$h={\left(\frac{2\pi N}{60}\right)}^{2}\cdot \left(\frac{2\pi x_{3}}{E_{f}}\right)\cdot \left(\frac{x_{1}^{4}}{4}-\frac{x_{2}^{4}}{4}\right),g=386.4,\Delta T_{\max}=50,W_{s}=101000\text{,}$$

$$h_{\min}=0.001,P_{\max}=1000$$

Table 13 records the results of PSMADE with PSO,^24^ NDE,^97^ TLBO,^99^ and GASO^101^ in solving this problem. It is seen that PSMADE obtains a solution of 19504.2206, the best result among all the above methods. It can be seen that a significantly improved performance of PSMADE on HTBP relative to the other algorithms.

**Table 13:** Results for each algorithm in the hydrostatic thrust bearing experiment

Algorithm	R	$R_{0}$	t	h	Best Cost
PSMADE	5.955780495	5.389013046	5.3587E-6	2.269655963	19504.2206
PSO	5.956868	5.389175	5.4021E-6	2.301546	19586.5788
NDE	5.955781	5.389013	5.3586E-6	2.269656	19506.0090
TLBO	5.955781	5.389013	5.3586E-6	2.269656	19506.0090
GASO	6.271000	12. 90100	5.6050E-6	2.938000	23403.4320

### Cantilever structure problem

**Figure 6:**
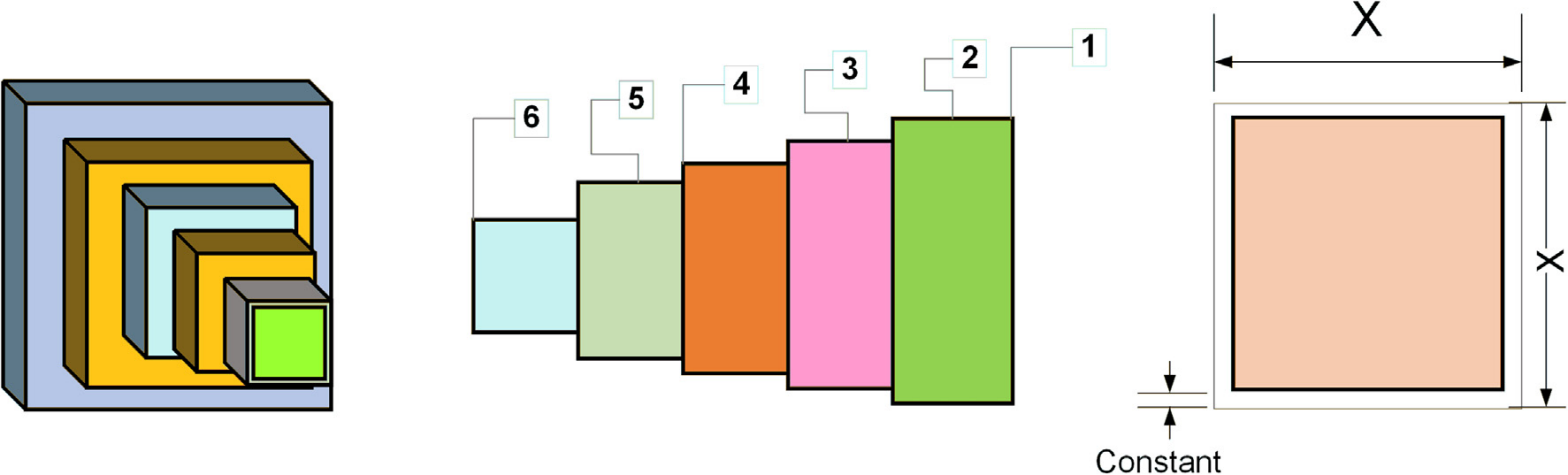
Structure of cantilever beam

This experiment aims to minimize the total mass of the cantilever arm while satisfying a set of constraints. The cantilever arm comprises five hollow square cross sections with the structure shown in Figure 6. Since the thickness of the cantilever arm material is fixed, only the six parameters illustrated in the figure need to be considered. The mathematical model of the problem is as follows.

**Consider**: $\vec{x}=\left[x_{1},x_{2},x_{3},x_{4},x_{5}\right]$

**Object**: $F{\left(\vec{x}\right)}_{\min}=0.6224\left(x_{1}+x_{2}+x_{3}+x_{4}+x_{5}\right)$

**Subject to**: $H_{1}\left(\vec{x}\right)=\frac{61}{x_{1}^{3}}+\frac{37}{x_{2}^{3}}+\frac{19}{x_{3}^{3}}+\frac{7}{x_{4}^{3}}+\frac{1}{x_{5}^{3}}\leq 1$

**Variable ranges**: $0.01\leq x_{1},x_{2},x_{3},x_{4},x_{5}\leq 100$

It is recorded in Table 14 the obtained results of PSMADE with SMA,^53^ MFO,^75^ MMA,^102^ CS,^103^ and GCA^58^ in solving this problem. It is observed that PSMADE obtains a solution of 1.3399564, the best consequence among all the above methods.

**Table 14:** Results for each algorithm in the cantilever structure problem experiment

Algorithm	$x_{1}$	$x_{2}$	$x_{3}$	$x_{4}$	$x_{5}$	Best Cost
**PSMADE**	**6.016834002**	**5.308420063**	**4.49384458**	**3.501811625**	**2.152749984**	**1.3399564**
SMA	6.017757	5.310892	4.493758	3.501106	2.150159	1.339957
MFO	5.9830	5.3167	4.4973	3.5136	2.1616	1.33998
MMA	6.0100	5.3000	4.4900	3.4900	2.1500	1.3400
CS	6.0089	5.3049	4.5023	3.5077	2.1504	1.33999
GCA	6.0100	5.3000	4.4900	3.4900	2.1500	1.3400

### Discussion and summary

SMA assigns a weight to each individual, endowing it with powerful space search capabilities. However, like other optimization algorithms, its performance tends to deteriorate in later stages. This paper presents a novel algorithm, PSMADE, which is an improved version of SMA based on DE and Powell mechanisms. Compared to SMA, PSMADE effectively addresses the issues of slow convergence speed, low accuracy, and premature convergence. Compared to other algorithms, PSMADE demonstrates greater competitiveness due to incorporating DE and Powell mechanisms, effectively balancing global exploration and local exploitation tendencies within the population. Based on the experimental results, PSMADE demonstrates superior comprehensive performance compared to the 29 MAs included in this study. Furthermore, compared to the original SMA, PSMADE exhibits enhanced capabilities in addressing complex problems such as unimodal, multimodal, hybrid, and composition functions.

To ensure the precise and dependable assessment of PSMADE’s competitiveness across diverse problem types, we have employed the CEC 2014 benchmark that encompasses unimodal, multimodal, hybrid, and composition functions as our testing foundation. On the CEC 2014 benchmark, we compared 14 original and 15 improved advanced algorithms using a combination of PSMADE with DE and Powell mechanisms. To ensure the rationality and scientific validity of the experimental results, we employed Wilcoxon signed rank test to verify their statistical significance. Among them, PSMADE exhibits the most remarkable enhancement in addressing unimodal and hybrid functions, thereby compensating for SMA’s limitations in tackling such problems.

To demonstrate the distinction between PSMADE and the original algorithm, we analyzed balance diversity for both PSMADE and SMA. The results indicate that PSMADE exhibits strong competitiveness. Furthermore, we applied PSMADE to solve four practical constraint engineering problems, including TCSD, BSDP, HTBP, and CSP. The experimental outcomes achieved by PSMADE in these four problems surpass those of all other algorithms involved in our comparison.

PSMADE outperforms 29 other methods in comparative experiments and solves four engineering problems. These rigorous and scientific experimental results demonstrate the superior comprehensive performance of PSMADE in tackling various complex problems. PSMAD achieves superior performance by leveraging the complementary strengths of DE and Powell mechanisms. During the search process, the mutation mechanism in DE enables rapid convergence toward optimal solutions. In later stages, the Powell mechanism combined with a taboo table enhances local utilization while avoiding excessive time complexity, thereby facilitating global optimization. Therefore, the proposed PSMADE also can try to be applied to more cases in future work, such as renal pathology image segmentation,^104^ global optimization,^105^^,^^106^ computer-aided medical diagnosis,^107^^,^^108^^,^^109^ cancer diagnosis,^110^ mental health prediction,^111^ and computational image analysis.^112^

However, despite ranking first in overall performance in the aforementioned experiments, PSMADE still requires further improvement compared to some advanced algorithms. As indicated by the experimental results presented in Table 8, PSMADE’s performance remains inadequate when dealing with certain functions. For instance, its comprehensive processing capability for unimodal functions F1-F3 is inferior to that of JADE, SHADE, and EBOwithCMAR. The overall processing performance of multimodal functions F4-F16 falls short compared to JADE, EBOwithCMAR, SHADE, LSHADE, CLPSO, and LSHADE_cnEpSi. Similarly, the comprehensive processing performance of hybrid functions is not on par with that of SHADE. Although the convergence speed and accuracy of PSMADE on various functions are comparable to those of other improved algorithms, it still exhibits weaknesses in premature convergence and population search stagnation when dealing with multimodal and hybrid functions. Therefore, further improvements are needed to enhance its ability to balance global exploration and local search.

### Conclusions and future works

This paper proposes PSMADE, which incorporates two effective mechanisms, DE and Powell, to address the issues of slow convergence, low accuracy, weak exploration ability, and susceptibility to local optima in SMA. The DE mechanism enhances communication among exceptional individuals, promotes population diversity, and significantly improves the algorithm’s global search ability. When combined with a taboo table, the Powell mechanism can explore deeper into the optimal solution space in the later stages of the algorithm, bringing it closer to achieving a global optimal solution and effectively improving its local utilization ability.

In order to comprehensively evaluate the performance of PSMADE, we not only compare it with SMA but also subject it to testing against 14 established MAs and 15 enhanced MAs on the CEC 2014 benchmark. The results demonstrate that PSMADE outperforms the original and 28 algorithms in the comparison, indicating its superior overall performance. Furthermore, we applied PSMADE to solve four practical constraint engineering problems (TCSD, BSDP, HTBP, and CSP), achieving higher solution accuracy than the other algorithms tested.

Although the PSMADE proposed in this study has demonstrated exemplary performance, it still exhibits limitations when applied to multi-objective problems, and its solution speed is slower than that of the original SMA. Therefore, our future development goal is to enhance its computational efficiency.

### Limitations of the study

The current study has several limitations. First, a comprehensive evaluation of the impact of the DE and Powell mechanisms introduced in the PSMADE algorithm on SMA has not been conducted, including qualitative analyses such as historical trajectory, balance, and diversity analysis. Existing studies have only provided preliminary comparisons of the performance between PSMADE, PSMA, SMADE, and SMA on the CEC 2014 benchmark. Second, although PSMADE exhibited excellent performance on the CEC 2014 benchmark, it still possesses certain limitations when dealing with multi-objective problems and requires further refinement. Additionally, in comparison to the original SMA algorithm, PSMADE exhibits slower solution speeds, necessitating further optimization to enhance computational efficiency. To address this concern, future research goals should focus on solving the limitations of PSMADE in handling multi-objective problems and further improving its computational efficiency. Parallel computing and distributed computing techniques can be considered to accelerate the solving process of the PSMADE algorithm.

## STAR★Methods

### Key resources table

**Table:** 

REAGENT or RESOURCE	SOURCE	IDENTIFIER
**Software and algorithm**		

SMA algorithm	Ali Asghar Heidari	https://aliasgharheidari.com/SMA.html and https://github.com/aliasgharheidaricom/Slime-Mould-Algorithm-A-New-Method-for-Stochastic-Optimization-
PSMADE algorithm	This paper	https://github.com/lvchuangchuang/PSMADE#psmade

### Resource availability

#### Lead contact

Further requests for information should be directed and will be handled by the lead contact, Yudong Zhang, email: yudongzhang@ieee.org.

#### Materials availability

This study did not generate new materials.

### Method details

The proposed methods, including slime mould algorithm, differential evolution algorithm, powell mechanism, will be explained in detail in this section.

#### Slime mould algorithm

The SMA, a novel swarm intelligence optimization algorithm, achieves global optimization by simulating the oscillatory predation behavior of individual slime mould. Slime moulds are unicellular organisms that thrive in warm, humid, and vegetation-rich environments, deriving their nutrients primarily from organic substances present in the external environment. During locomotion, the anterior end of the slime mould expands into a fan-like shape, giving rise to an interconnected network of veins composed of varying thicknesses. By sensing airborne chemical cues, slime moulds can locate sources of nutrients.

The biological oscillators found in slime moulds can generate propagating waves that regulate cytoplasmic flow and velocity. When a slime mould's vein is situated near its food source, the biological oscillator produces a propagation wave that amplifies cytoplasmic flow within the vein, resulting in an increased flow rate and width. Conversely, as the slime mould vein moves away from the food source, its internal cytoplasmic flow and width decrease. By utilizing positive and negative feedback generated by a propagation wave based on biological oscillation, the slime mould can establish an optimal path to connect with food in a superior manner. The three main logical steps of SMA include approaching, wrapping around, and grasping onto food. The primary procedures of SMA are outlined as follows.

##### Approach food

The slime moulds have to approach the physical object first when feeding, and Li proposed the following mathematical model to describe this behavior, as shown in Equation 1.

(Equation 1) $$\vec{X\left(t+1\right)}=\left\{ \begin{array}{l} \vec{X_{b}\left(t\right)}+\vec{vb}\cdot \left(\vec{W}\cdot \vec{X_{A}\left(t\right)}-\vec{X_{B}\left(t\right)}\right),r<p\\ \vec{vc}\cdot \vec{X\left(t\right)},r\geq p \end{array}\right.$$

where t is the number of current iterations; $\vec{X\left(t\right)}$ denote the positions of the slime moulds at the t th iteration; $\vec{X_{b}\left(t\right)}$ represents the position of the current optimal individual; $\vec{X_{A}\left(t\right)}$, $\vec{X_{B}\left(t\right)}$ are the positions of two randomly selected individuals in the current mid-population, where A≠B; $\vec{W}$ is the quality of the slime bacteria individuals, representing their fitness weight coefficients; p=tanh|S(i)−DF| is the control parameter for the algorithm to balance the exploitation and exploration ability; $\vec{vb}$ and $\vec{vc}$ are control parameters, $\vec{vb}\in \left[-a,a\right]$, $\vec{vc}\in \left[-1,1\right]$, where $a=\mathrm{arctanh}\left(-\left(\frac{t}{MaxFEs}\right)+1\right)$, $\vec{vb}$ and $\vec{vc}$ oscillate between the ranges and gradually approach zero; N is the population number of individuals; S(i) denotes the fitness value of the i th individual, i∈1,2,...,N; DF denotes the optimal fitness value found in all iterations of the population up to now; MaxFEs is the maximum number of iterations; r is a random number between [0,1].

The formula for the parameter $\vec{W}$ in Equation 1 is shown in Equation 2 shown.

(Equation 2) $$\vec{W\left(SmellIndex\left(i\right)\right)}=\left\{ \begin{array}{l} 1+r\cdot \log\left(\frac{bF-S\left(i\right)}{bF-wF}+1\right),condition\\ 1-r\cdot \log\left(\frac{bF-S\left(i\right)}{bF-wF}+1\right),others \end{array}\right.$$

where condition denotes the upper half of the fitness of the individual in the population and others denotes the remaining slime mould individuals; r refers to a random number between [0,1]; bF denotes the best fitness value in the current population iteration; wF denotes the worst fitness value in the current population iteration; SmellIndex(i)=sort(S) is the ranked sequence of fitness values.

##### Wrap food

During the wrapping phase of food, the constriction pattern of slime mould vein tissue can be mathematically modeled. As the concentration of food increases, biological oscillators within the slime mould generate stronger propagation waves, thereby increasing vein width. Equation 1 describes slime moulds' positive and negative feedback mechanisms between food concentration and mucus vein width. In regions with higher food concentrations, individuals will search deeper due to food stimulation, while some individuals will be separated to explore other areas for global optimization. The mathematical model of slime mould population renewal location is presented in Equation 3.

(Equation 3) $$\vec{X\left(t+1\right)}=\left\{ \begin{array}{l} \text{rand}\cdot \left(UB-LB\right)+LB,rand<z\\ \vec{X_{b}\left(t\right)}+\vec{vb}\cdot \left(\vec{W}\cdot \vec{X_{A}\left(t\right)}-\vec{X_{B}\left(t\right)}\right),r<p\\ \vec{vc}\cdot \vec{X\left(t\right)},r\geq p \end{array}\right.$$

where rand, r are random numbers between [0,1]; UB and LB are the upper and lower bounds of the search region, respectively; and z is a custom parameter. In the original paper of SMA, the authors experimentally determined that when can better balance the exploration and development phases when z=0.03, so the same parameter settings are adopted in this paper.

##### Grabble food

In this stage, the propagation wave of the biological oscillator within the slime mould exerts an influence on both cytoplasmic flow rate and velocity. By utilizing $\vec{vb}$, $\vec{vc}$, and $\vec{W}$ to simulate changes in static width and oscillation frequency of the slime mould, it is able to gradually approach areas with low food concentration while quickly locating and capturing food in regions with high concentrations.

Figure S1 illustrates the general logic of SMA.

#### Differential evolution algorithm

The DE was introduced by Storn and Price in 1997.^21^ DE models the cooperation and competition among individuals within a population to achieve global optimization, with its logical steps consisting of mutation, crossover, and selection. Specifically, the main steps of DE are as follows.

##### Variation operator

One individual is selected randomly from the population to undergo mutation, while the vector difference between two randomly chosen individuals is scaled and integrated with the position vector of the mutated individual. The mathematical model for this mutation operation is presented in Equation 4.

(Equation 4) $$\vec{V_{i}\left(t+1\right)}=\vec{X_{r1}\left(t\right)}+F\cdot \left(\vec{X_{r2}\left(t\right)}-\vec{X_{r3}\left(t\right)}\right)$$

where t is the number of current iterations; r1, r2, and r3 are random integers between [1,N], and the three are not equal. F is the mutation operator, F∈[0,2], and the value of F can affect the population diversity, where the larger the value of F, the population diversity is greater, and the algorithm converges more slowly, and vice versa, the speed of convergence is faster.

##### Cross operation

The cross operation is to cross the variant individual $\vec{V_{i}\left(t+1\right)}$ obtained from the mutation operation with the current individual $\vec{X_{i}\left(t\right)}$ to obtain the next-generation candidate $\vec{U_{i}\left(t+1\right)}$ of the current individual. The mathematical model of the crossover operation is shown in Equation 5.

(Equation 5) $$\vec{U_{i}^{j}\left(t+1\right)}=\left\{ \begin{array}{l} \vec{V_{i}^{j}\left(t+1\right)},rand\leq P_{CR},\mathrm{ran}d_{j}=j\\ \vec{X_{i}^{j}\left(t\right)},rand>P_{CR},\mathrm{ran}d_{j}\neq j \end{array}\right.,j=1,2,...,\dim$$

where $P_{CR}$ is a random number between [0,1], representing the crossover probability; rand is a random number between [0,1]; $\mathrm{ran}d_{j}$ is a random integer between [1,dim], and dim is the dimension of the problem.

##### Select operation

DE employs a greedy selection strategy to determine the next generation of individuals, selecting the superior individual between the candidate individual $U_{i}\left(t+1\right)$ and the current individual $X_{i}\left(t\right)$ generated through mutation and crossover operations. The mathematical model for this selection process is presented in Equation 6.

(Equation 6) $$\vec{X_{i}\left(t+1\right)}=\left\{ \begin{array}{l} \vec{U_{i}\left(t+1\right)},f\left(\vec{U_{i}\left(t+1\right)}\right)<f\left(\vec{X_{i}\left(t\right)}\right)\\ \vec{X_{i}\left(t\right)},else \end{array}\right.$$

where f as the function to calculate the individual fitness value.

#### Powell mechanism

The Powell search method, also known as the direction acceleration method. It employs conjugate directions to expedite convergence, circumvents intricate gradient operations, and possesses formidable search capabilities. Although this method has been around for a long time, due to its significant advantages in local search, many scholars have applied it to improve various types of optimization problems and achieved excellent results.^113^^,^^114^ In SMA, we incorporate the Powell mechanism later in the iteration to enable deeper local exploration by the population and enhance the likelihood of converging toward a globally optimal solution.

Powell’s method consists of three stages: a basic search, an accelerated search, and an adjusted search. The basic search starts from an initial position and performs a 1D polar search along a known direction to generate a new position vector. The accelerated search computes the difference between two adjacent position vectors to obtain the search direction closer to the target, which is replaced by the original search direction. Finally, the adjusted search is performed by replacing one of the known search directions with the connection directions obtained in the accelerated search phase to form a new set of directions for the next iteration. This process is repeated, and the extreme value search of the target is finally achieved.

The specific implementation of Powell’s algorithm is as follows.

**Step1**: Select the initial data. Given an initial node $u^{\left(0\right)}$, an allowable error ε(ε>0), and dim linearly independent initial search directions $d^{\left(i\right)}$ where i=1,2,...,dim, dim is the dimension of the function and let k=0.

**Step2**: Perform the basic search. Calculate $\alpha_{i}$ by Equation 7, and then perform a one-dimensional search from $u^{\left(0\right)}$, along $d^{\left(0\right)},d^{\left(1\right)},...,d^{\left(\dim-1\right)}$ to obtain new base points $u^{\left(1\right)},u^{\left(2\right)},...,u^{\left(\dim\right)}$ . The mathematical expression of this step is as indicated in Equation 8.

(Equation 7) $$f\left(u^{\left(i\right)}+\alpha_{i}d^{\left(i\right)}\right)=\min\left(f\left(u^{\left(i\right)}+\alpha_{i}d^{\left(i\right)}\right)\right)$$

(Equation 8) $$u^{\left(i+1\right)}=u^{\left(i\right)}+\alpha_{i}d^{\left(i\right)},i=0,1,...,\dim-1$$

where α and $\alpha_{i}$ are the steps of the search, if $\alpha_{i}$ is negative, it means that the linear search is performed on the real number axis; if i<dim−1, set i=i+1 and continue to execute **Step2**; otherwise, run **Step3**.

**Step3**: Perform an accelerated search. Calculate $d^{\left(\dim\right)}$ by $d^{\left(\dim\right)}=u^{\left(\dim\right)}-u^{\left(0\right)}$, determine whether $\left\|d^{\left(\dim\right)}\right\|\leq \varepsilon$ holds, if it holds, then $u^{\left(\dim\right)}$ is the approximate minimal value solution and the calculation is finished; otherwise, skip and execute **Step4**.

**Step4**: Calculate the index tl determined by the maximum decline using Equation 9. Determine whether Equation 10 holds, if it does, it indicates that $d^{\left(0\right)},d^{\left(1\right)},...,d^{\left(\dim-1\right)}$ is still linearly irrelevant and the direction of the next round of search remains the same, so let $u^{\left(0\right)}=u^{\left(\dim\right)}$, k=k+1, step to **Step2**; otherwise, it indicates that $d^{\left(0\right)},d^{\left(1\right)},...,d^{\left(\dim-1\right)}$ is linearly irrelevant and step to **Step5**.

(Equation 9) $$f\left(u^{\left(tl\right)}\right)-f\left(u^{\left(tl+1\right)}\right)=\max_{0\leq i\leq \dim-1}\left\{f\left(u^{\left(i\right)}\right)-f\left(u^{\left(i+1\right)}\right)\right\}$$

(Equation 10) $$f\left(u^{\left(0\right)}\right)-2\cdot f\left(u^{\left(\dim\right)}\right)+f\left(2\cdot u^{\left(\dim\right)}-u^{\left(0\right)}\right)\geq 2\cdot \left(f\left(u^{\left(tl\right)}\right)-f\left(u^{\left(tl+1\right)}\right)\right)$$

**Step5**: Perform adjusted search. Since Equation 10 does not hold, indicating that $d^{\left(0\right)},d^{\left(1\right)},...,d^{\left(\dim-1\right)}$ is linearly correlated, let $d^{\left(tl+i\right)}=d^{\left(tl+i+1\right)}$, i=1,2,...,dim−tl−1 to ensure that the newly generated search direction is linearly uncorrelated. Then use Equation 7 to compute $\alpha_{\dim}$, let $u^{\left(0\right)}=u^{\left(\dim+1\right)}=u^{\left(\dim\right)}+\alpha_{\dim}d^{\left(\dim\right)}$ and k=k+1, and skip to **Step2**.

#### Proposed methodology

The pseudocode of PSMADE is presented in Algorithm 1. Even though the original SMA outperforms other optimizers such as PSO, ACO, and DE according to Li et al.'s experimental results,^32^ it still suffers from the issue of getting trapped in local optima when dealing with multimodal, composition, and other benchmark functions. We propose incorporating DE and Powell mechanisms to address these limitations and enhance SMA's global optimization capability.

During the algorithm development stage, the DE mechanism was successfully integrated into the SMA to enhance information exchange among dominant individuals in the population and prevent premature convergence to local optima. Introducing the DE mechanism enables individuals in the population to explore and utilize the search space more effectively, thereby enhancing the algorithm's global search ability. In later iterations, the Powell mechanism can conduct a thorough search near the optimal solution, promoting convergence toward the target solution. To minimize the time cost of the Powell mechanism, taboo tables are implemented to prevent repetitive excavation at the same location.

The PSMADE algorithm comprises four main components: population initialization, DE mechanism, SMA population position update mechanism, and Powell mechanism. Initially, the population is initialized as a set of randomly generated individuals. Subsequently, it undergoes iterations through the DE mechanism to facilitate information exchange between individuals and search space exploration. In later stages of the algorithm, the Powell mechanism is introduced to perform an in-depth search near more optimal solutions. This combination mode enables PSMADE to overcome limitations posed by local optima and converge towards target solutions with superior global optimization abilities. The steps are as follows.

**Step 1:** Initialize the population. PSMADE generates the initial slime mould population using Equation 11.

(Equation 11) $$\vec{X_{i}}=rand\cdot \left(\vec{UB}-\vec{LB}\right)+\vec{LB},i=1,2,...,N$$

where N is the size of the population, $\vec{X_{i}}$ is the i th individual in the population, rand refers to a random number in the range [0,1], and UB and LB denote the upper and lower bounds of the search region, respectively.

**Step 2:** DE mechanism. Firstly, using the optimal individual position $\vec{X_{i}^{pb}\left(t\right)},\left(i=1,2,...,N\right)$ searched by each individual so far, update the position according to Equations 4, 5, and 6 to generate the new population position $\vec{X_{i}^{DE}\left(t\right)},\left(i=1,2,...,N\right)$. Then, using the greedy selection strategy, compare $\vec{X_{i}^{DE}\left(t\right)}$ with $\vec{X_{i}\left(t\right)}$ and retain the individual with the better fitness value to continue the subsequent operation. The retention process is shown in Equation 12.

(Equation 12) $$\vec{X_{i}\left(t\right)}=\left\{ \begin{array}{l} \vec{X_{i}^{DE}\left(t\right)},f\left(\vec{X_{i}^{DE}\left(t\right)}\right))<f\left(\vec{X_{i}\left(t\right)}\right)\\ \vec{X_{i}\left(t\right)},else \end{array}\right.$$

where t is the current number of iterations and f is the function that calculates the fitness value of the individual.

**Step 3:** SMA's population position update mechanism. Slime mould individuals in the population update their positions according to Equations 1, 2, and 3.

**Step 4:** Powell mechanism. When the current number of iterations t>0.8×MaxFEs, determine whether the currently found best individual position $\vec{X_{b}\left(t\right)}$ is in the taboo table, if not, put $\vec{X_{b}\left(t\right)}$ into the taboo table, then set $\vec{X_{b}\left(t\right)}$ as the initial position and ε as $10^{-6}$, get a new position $\vec{X^{powell}\left(t\right)}$ according to Equations 7, 8, 9, and 10, and update it according to Equation 13; conversely, execute **Step 5**.

(Equation 13) $$\vec{X_{b}\left(t\right)}=\left\{ \begin{array}{l} \vec{X^{powell}\left(t\right)},f\left(\vec{X^{powell}\left(t\right)}\right)<f\left(\vec{X_{b}\left(t\right)}\right)\\ \vec{X_{b}\left(t\right)},else \end{array}\right.$$

**Step 5:** Repeat the execution of **Step 2-Step 4** until the current solution is the optimal solution or stop the loop when the termination condition is satisfied and output the optimal solution.

Figure S2 illustrates the overarching logic of PSMADE, while Figure S3 presents a flowchart detailing the execution of the algorithm for enhanced comprehension. These enhancements and integrations have given PSMADE superior global search and optimization capabilities when tackling multimodal, composition, and other benchmark functions.

**Algorithm 1:** Pseudo-code for PSMADE

Input: Parameters N, dim, MaxFEs, etc;
Output: Optimal solution;
**Initialize** the positions of slime moulds $\vec{X_{i}}\left(i=1,2,...,N\right)$;
**Initialize**taboo = [ ];
**Initialize** some of the remaining variable parameters;
**While**t<MaxFEs
**For**i=1 to N
**Fix** agents that exceed search boundaries;
**Calculate** the fitness of the agent by the objective function;
**Update** the best search agent $\vec{X_{b}}$ and bestFitness;
**End For**
**Get** the positions of $\vec{X^{DE}}$ by Equations 4, 5, and 6;
**Update** the positions of agents $\vec{X}$ by Equation 12;
**Sort** in ascending order by fitness;
**Update**bestFitness, bF, wF;
**Calculate** the weights $\vec{W}$ by Equation 2;
**For**i=1 to N
**Calculate** parameters p, $\vec{vb}$, $\vec{vc}$;
**Update** position of agent by Equation 3;
**End For**
**If**t<0.8×MaxFEs
**If**$\vec{X_{b}}$ not in taboo
**Add**$\vec{X_{b}}$ into taboo;
**Get** the position of $\vec{X^{powell}}$ by Equations 7, 8, 9, and 10;
**Update** the $\vec{X_{b}}$ by Equation 13;
**End If**
**End If**
**Let**t=t+1;
**End While**
Output the best solution, $\vec{X_{b}}$

### Quantification and statistical analysis

A detailed description of the statistical methods is presented in the results and discussions section of the following subsections: Verification of the mechanisms, Comparison with conventional metaheuristic algorithms, Comparison with improved algorithms, and Experiments on engineering benchmarks. All experiments were conducted on the same hardware and MATLAB R2014b software environment to ensure the objectivity and fairness of the results. In the context of global optimization problems, including the PSMADE algorithm, these methods utilize statistical measures such as the average (Avg) and standard deviation (Std) of the objective function to evaluate their performance. The average value is a key indicator for assessing solution quality, where lower values indicate better global optimization search capabilities and solution quality. The standard deviation, on the other hand, measures the spread of solutions, with a lower standard deviation indicating better stability of the algorithm. To determine if the proposed improvements are statistically significant compared to other methods, we employed a non-parametric statistical test, specifically the Wilcoxon signed-rank test, with a significance level set to 0.05. The symbols "+/=/-" indicate whether the proposed algorithm performs better, equal, or worse compared to the other methods.

The detailed statistical results of the global optimization experiments can be found in Tables 2, 3, 5, 6, 8, and 9, as well as Figures 3, 4, and 5. In the engineering benchmark experiments, we also performed statistical analysis on the application of the PSMADE algorithm along with other metaheuristic algorithms, Constraint Correction, and Mathematical Optimization to solve real-world engineering problems. The specific results are provided in Tables 10, 12, 13, and 14. Furthermore, all statistical details are provided and explained in the manuscript.

## Data Availability

• The dataset utilized in this study is accessible online and will be shared by the primary contact upon request. All original code developed for this study has been uploaded to the website https://aliasgharheidari.com/ or GitHub and has been publicly available since the date of publication. The key resources table includes links to the code and DOIs. • The data presented in this paper will be made available by the primary contact upon request. • The original code is not reported in this paper. • Any additional information required for reanalyzing the data reported in this paper can be obtained from the designated contact person upon request.
