# Supplementary material for: Advanced slime mould algorithm incorporating differential evolution and Powell mechanism for engineering design
Source: iScience. 2023 Aug 28;26(10):107736. doi: 10.1016/j.isci.2023.107736 (PMC10558746; doi:10.1016/j.isci.2023.107736)
Supplement: Document S1. Figures S1–S3 [file mmc1.pdf]

iScience, Volume *26*

## Supplemental information

# Advanced slime mould algorithm incorporating differential evolution and Powell mechanism for engineering design

**Xinru Li, Zihan Lin, Haoxuan Lv, Liang Yu, Ali Asghar Heidari, Yudong Zhang, Huiling Chen, and Guoxi Liang**

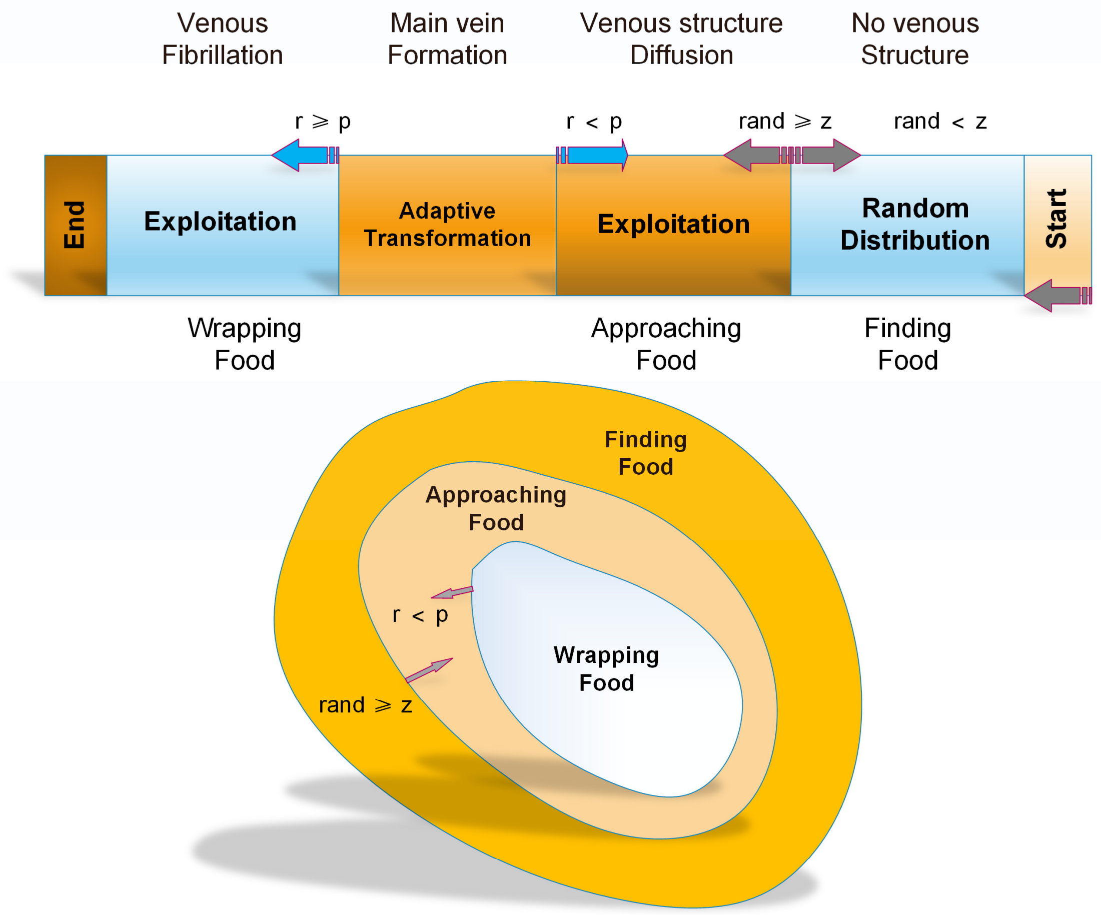


**Figure S1**. Different phases of the SMA, Related to STAR Methods

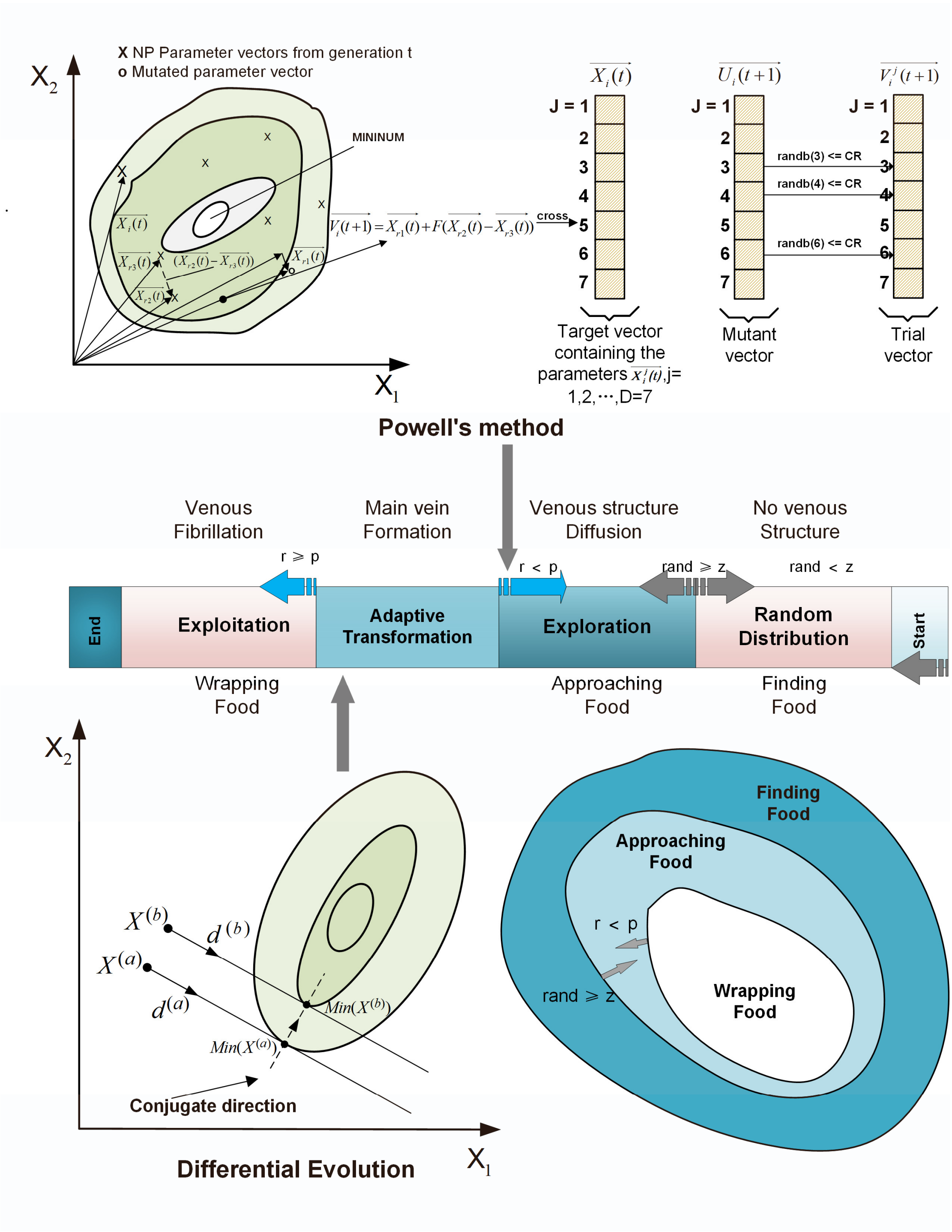


**Figure S2**. Overview logic of PSMADE, Related to STAR Methods

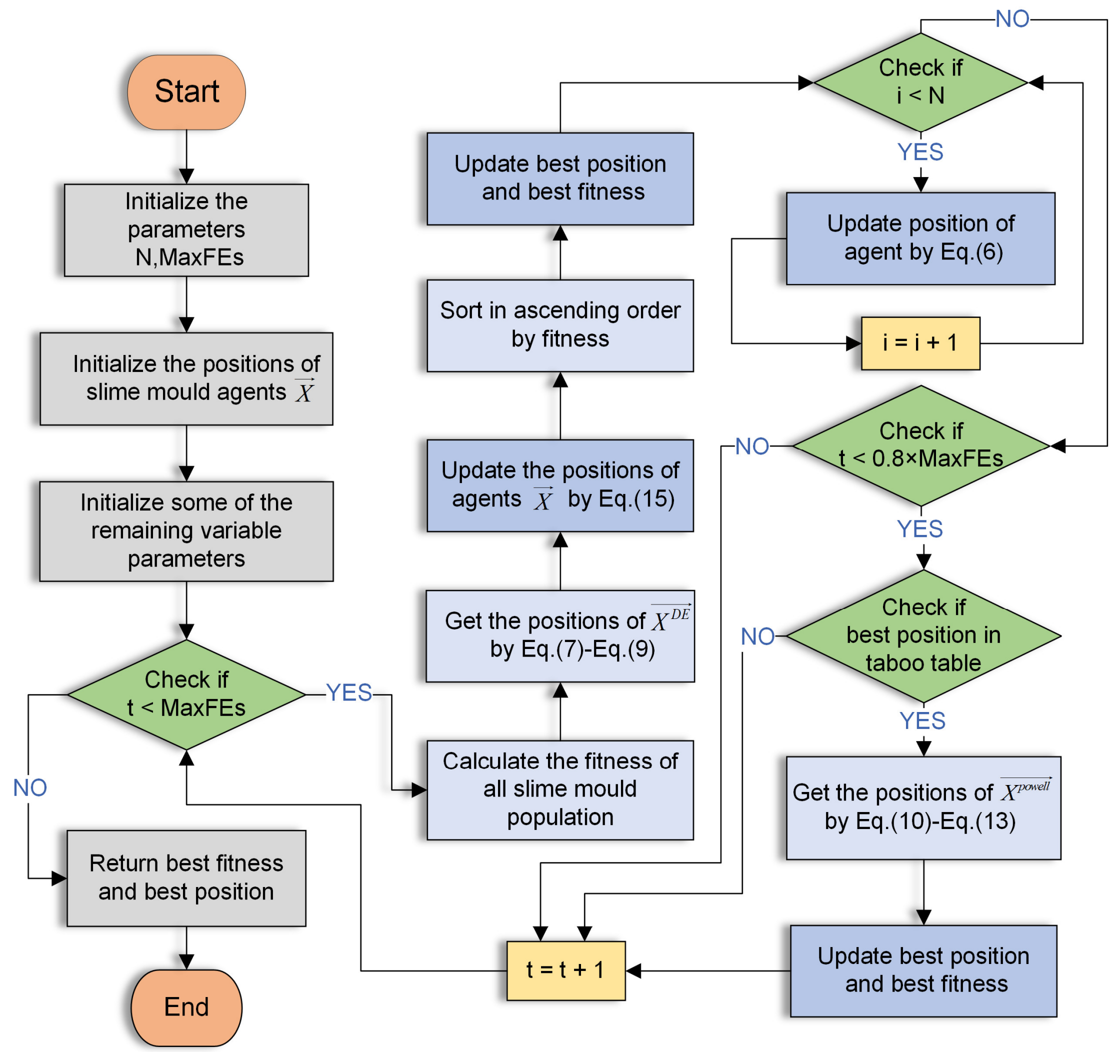


**Figure S3**. Flowchart of PSMADE, Related to STAR Methods
